# *Anopheles aquasalis* Infected by *Plasmodium vivax* Displays Unique Gene Expression Profiles when Compared to Other Malaria Vectors and Plasmodia

**DOI:** 10.1371/journal.pone.0009795

**Published:** 2010-03-22

**Authors:** Ana C. Bahia, Marina S. Kubota, Antonio J. Tempone, Waleria D. Pinheiro, Wanderli P. Tadei, Nágila F. C. Secundino, Yara M. Traub-Csekö, Paulo F. P. Pimenta

**Affiliations:** 1 Laboratório de Biologia Molecular de Parasitas e Vetores, Instituto Oswaldo Cruz, Fiocruz, Rio de Janeiro, Rio de Janeiro, Brazil; 2 Laboratório de Malária e Dengue, Instituto Nacional de Pesquisas da Amazônia, Manaus, Amazonas, Brazil; 3 Laboratório de Entomologia Médica, Instituto René Rachou, Fiocruz, Belo Horizonte, Minas Gerais, Brazil; Federal University of São Paulo, Brazil

## Abstract

Malaria affects 300 million people worldwide every year and is endemic in 22 countries in the Americas where transmission occurs mainly in the Amazon Region. Most malaria cases in the Americas are caused by *Plasmodium vivax*, a parasite that is almost impossible to cultivate *in vitro*, and *Anopheles aquasalis* is an important malaria vector. Understanding the interactions between this vector and its parasite will provide important information for development of disease control strategies. To this end, we performed mRNA subtraction experiments using *A. aquasalis* 2 and 24 hours after feeding on blood and blood from malaria patients infected with *P. vivax* to identify changes in the mosquito vector gene induction that could be important during the initial steps of infection. A total of 2,138 clones of differentially expressed genes were sequenced and 496 high quality unique sequences were obtained. Annotation revealed 36% of sequences unrelated to genes in any database, suggesting that they were specific to *A. aquasalis.* A high number of sequences (59%) with no matches in any databases were found 24 h after infection. Genes related to embryogenesis were down-regulated in insects infected by *P. vivax*. Only a handful of genes related to immune responses were detected in our subtraction experiment. This apparent weak immune response of *A. aquasalis* to *P. vivax* infection could be related to the susceptibility of this vector to this important human malaria parasite. Analysis of some genes by real time PCR corroborated and expanded the subtraction results. Taken together, these data provide important new information about this poorly studied American malaria vector by revealing differences between the responses of *A. aquasalis* to *P. vivax* infection, in relation to better studied mosquito-*Plasmodium* pairs. These differences may be important for the development of malaria transmission-blocking strategies in the Americas.

## Introduction

Malaria affects 300 million people worldwide every year. Among the endemic countries 22 are in the Americas, where transmission occurs mainly in the Amazon region. Brazil had an estimated 1.4 million malaria cases in 2006, over half of the total for the Americas, while Colombia had an estimated 408,000 cases (“WHO/HTM/GMP/2008.1”). The malaria parasites that circulate in this region are *Plasmodium falciparum*, *Plasmodium vivax* and *Plasmodium malariae* while the main vectors are *Anopheles darlingi*, *Anopheles aquasalis* and some species of the *Anopheles albitarsis* complex [1]. The ineffectiveness of vaccines, the resistance of *Plasmodium* to drugs and of insects to insecticides indicates the need for discovering new strategies to combat this disease. In some A*nopheline* species studies aiming at blocking malaria transmission have been successful [2].

The parasite responsible for the majority of malaria cases in the Americas is *P. vivax*. The mosquito *A. aquasalis* is an important American malaria vector that breeds in brackish coastal marsh habitats from Nicaragua to Southern Brazil [3]–[5]. The *Plasmodium sp.* life cycle in the insect vector is very complex. During 14 to18 days the parasite passes through various stages of development, and interacts with different tissues of the insect. The study of the reproduction and development of these parasites in their vector is essential for the development of new malaria control strategies. However, almost all previous studies are based on African and Asian anopheline species such as *Anopheles gambiae* infected with *P. falciparum* or *Plasmodium berghei* and *Anopheles stephensi* infected with *P. berghei* [6]–[7].

The main goal of this study is to analyze the effect of *P. vivax* infection on *A. aquasalis* gene expression. Due to the lack of a practical continuous cultivation system for *P. vivax* [8] insects used in these studies were fed on blood from *P. vivax* malaria patients.

Different subtraction mRNA libraries were constructed and analyses of these libraries revealed numerous mosquito genes that are up and down–regulated by infection. The analysis of these parasite induced *A. aquasalis* genes should lead to a better understanding of this vector-parasite relationship and to the identification of targets for blocking malaria transmission.

## Results and Discussion

### Subtraction experiments

Based on the precedent from anopheline studies [6]–[7] that *Plasmodium* infection can induce genes responsible for immunity, as well as more general aspects of stress, we constructed mRNA subtraction libraries with the aim of identifying genes regulated by this challenge. *A. aquasalis* were infected with *P. vivax* through artificial feedings with blood of malaria patients. All infected insect samples utilized in this study were tested for *P. vivax* infection by PCR and all of them amplified the expected 84 bp band (Figure S1).

Subtraction cDNA libraries were constructed for these studies from RNAs obtained from insects at 2 and 24 hours post-feeding on uninfected blood (F) or on infected blood (I). Four libraries were constructed using cDNAs from 2 and 24 hours infected minus non-infected insects (named 2hI-F and 24hI-F, respectively) and 2 and 24 hours non-infected minus infected insects (2hAF-I and 24hAF-I, respectively). The 2 and 24 hour timepoints were chosen for library generation since they represent the first stages of infection prior to parasite differentiation into oocysts, and thus should provide data most relevant to development of transmission blocking strategies. At 2 hours after infection (AI) the gametocytes differentiate and fertilization and zygote formation occur, and at approximately 24 hours AI the ookinetes pass through the midgut epithelium, a crucial and traumatic step in infection.

The amplicons obtained from these libraries (Figure S2) were cloned and 2,138 cDNA fragments were sequenced. A total of 1,047 high quality sequences were clusterized generating 496 unique fragments (Table 1). For protein function prediction, the sequences were compared to insects and plasmodia sequences, to curated databases and databases of conserved domains and orthologs. Of the total sequences, an elevated percentage had best matches with insect (98%) databases. A low number of sequences presented hit with *Plasmodium* database, and since these were related to very conserved sequences, they could be of mosquito origin. This absence of *Plasmodium* sequences can be explained by the low parasite load observed in this natural vector-parasite pair or the early times of infection, before sporozoites amplification.

**Table 1:** Data of subtraction libraries.

Library	Average length of inserts	Number of Sequences	Sequences with high quality	Unique Sequences
**2hF-I**	335 bp	606	285	180
**2hI-F**	236 bp	521	358	179
**24hF-I**	245 bp	376	234	73
**24hI-F**	365 bp	635	170	64
**Total/Average**	295 bp	2138	1047	496

F-I: libraries of cDNA after feeding minus after infection and I-F: libraries of cDNA after infection minus after feeding. h–Hours of feeding or infection.

bp: base pair.

**Figure 1:**
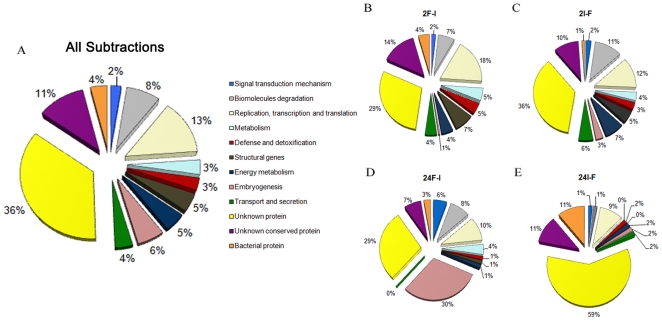
Overview of the functional breakdown of subtraction library ESTs based on blast similarities. Pie charts indicating the relative proportions of gene groups in all libraries (A), 2hF-I (B), 2hI-F (C), 24hF-I (D) and 24hI-F (E) libraries. Deduced proteins were grouped by similarity in predicted biological functions. The functional categories of genes and their corresponding colors are indicated.

After annotation, sequences were divided into 13 different categories based on their biological functions (Figure 1A, Tables S1, S2, S3, S4). Altogether, 36% of the sequences did not present hits in any database. These could be specific for *A. aquasalis* or represent untranslated regions of genes poorly conserved among species. Also, 11% of the sequences coded for unknown conserved proteins, normally related with other mosquito sequences which might represent conserved genes useful in ample spectrum control strategies.

One major category of genes (59%) identified in the 24hI-F library codes for unknown proteins (Figure 1E). These genes may play as-of-yet unknown roles in the interaction of the mosquito with the parasite. Another important difference between the 24hI-F and the 24AF-I libraries was the number of embryogenesis-related genes, almost entirely composed by vitellogenin ESTs, which were down-regulated by the insect infection. While 30% of the sequences from the 24hF-I library were embryogenesis related, only 1.6% were found in the 24hI-F library (Figures 1D and E). Interestingly, some authors have described interference of *Plasmodium* infection on the mosquito reproductive fitness [9], which is consistent with these results. This may be due to the cost of building an immune response that leads to the functional tradeoff between mating and immunity [10]. Another possible explanation is a metabolic deficiency generated by the down modulation of genes related to energy metabolism. As an example, proline oxidase transcripts were only observed in the 2hF-I library (Table S1) [11]. Alternatively, parasite nutrient acquisition or expression alteration of some digestive enzymes, as observed for the chymotrypsin-like serine protease discussed below, could inhibit blood digestion and absorption of nutrients leading to decreased number of embryogenesis related genes expression.

Unexpectedly, only 3% of all ESTs (15 sequences) were related to immunity (Figure 1A), and no differences were observed in numbers of immune related genes among the libraries (Figures 1B-E). A feeble response to the parasite, as we report here, was seen in *A. stephensi* infected with *P. berghei* [6]. In contrast, a strong immune response was seen in the natural vector-parasite pair *A. gambiae*-*P. falciparum* [7]. The lack of a strong immune response of *A. aquasalis* in the early steps of *P. vivax* infection could be responsible for the success of colonization and transmission of this malaria parasite. These differences in the biology of Old and New World parasite-vector interactions could well be due to genetic/evolutionary factors since, albeit belonging to the same genus, these species have evolved in distant parts of the world, submitted to very different environmental pressures. Still, we cannot exclude completely the possibility of the subtraction approach missing some immune genes. Future analyses using more sensitive mRNA abundance assays will be used to address this issue when genome sequences become available.

### Validation of cDNA subtractions and analyzes of gene expression

To validate differential expression results a subset of the genes identified in the subtraction libraries were selected for analysis by real time PCR (RTPCR). In some cases, the expression of some of these genes was investigated in relation to blood-feeding, development, and comparing males and females. The majority of RTPCR experiments corroborated the subtraction library results. The few inconsistencies observed are related to the nature of the subtraction screening methodology. Nevertheless, these did not invalidate this approach, but rather confirmed its utility as a technique to identify differentially expressed sequences and rare transcripts [12].

### Cellular structural genes

**Figure 2:**
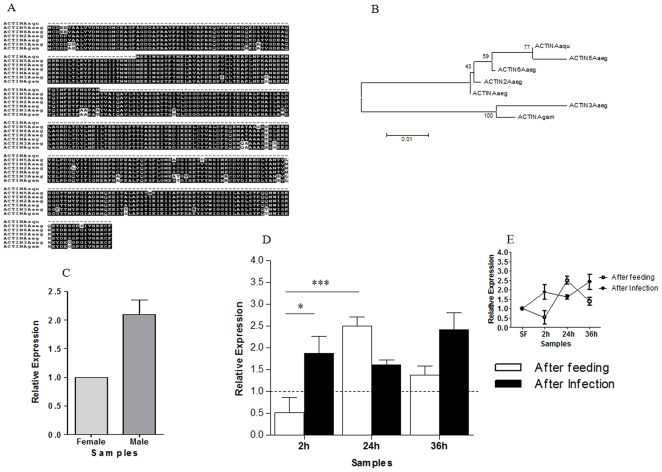
Characterization of actin induction in mosquitoes fed on sugar, blood, or infected blood. A: Multiple alignment of mosquito actin sequences. B: The phylogenetic tree was constructed using neighbor-joining. C–E: Expression levels of actin determined by RTPCR following various feeding regimens. C: Sugar-fed males and females, D and E: sugar-fed (dotted line and SF), blood-fed (AF) and *P. vivax* infected (AI) females. h–hours. Accession numbers of actin sequences from: *A. aquasalis* (Aaqu) (GR486917); *A. aegypti* (Aaeg) (ACTIN - EMBLEAT47188.1, ACTIN2 - EMBLAAQ24506.1, ACTIN3 - EMBLAAQ24507.1, ACTIN5 - EMBLAAY81972.1, and ACTIN6 - EMBLAAZ31061.1); and *A. gambiae* (Agam) (ACTIN - EMBLCAJ14142.1). +−: s.e.m.; * *p*<0.05, ** 0.03>*p*>0.01, *** *p*<0.01.

Among the genes related to cellular structure, we chose to characterize the expression of actin. Four actin ESTs were identified. Phylogenetic approaches showed that one of these (GR486917) was more closely related to actin5 of *Aedes aegypti* (Figures 2A and B). The expression of this gene was higher in males than sugar-fed (SF) females (Figure 2C). In females, expression increased after blood ingestion at 24 hours after feeding (AF) and returned to almost basal levels at 36 hours AF (Figures 2D and E), which may be due to the distention of the insect abdomen caused by ingestion of almost three parts of its weight in blood. Also, actin expression was higher at 2 h AI when compared with 2 h AF. This result is in agreement with the subtraction result, where all actin transcripts were found in 2hI-F library (Table S2). Most importantly, infection of mosquitoes with *P. vivax* increased slightly the expression of this gene between 2 and 36 hours AI (Figures 2D and E). This coincides with the passage of the *Plasmodium* ookinete between epithelial cells and establishment in the basal lamina of the epithelium. This parasite route has been already shown to disrupt and reorganize actin filaments, with expulsion of the infected cells to the midgut lumen, in other mosquito-*Plasmodium* models [13], [14]. We suggest that this phenomenon can be the cause for the increase in actin mRNA levels in *A. aquasalis* after *P. vivax* infection. The actin gene, which has been widely used as a constitutive control in quantitative expression experiments [15], in *A. aquasalis* varied with feeding and infection, showing that this gene is not useful to normalize RTPCR, as had already been shown for *A. gambiae* [16].

### Digestive enzymes

Based on the importance of digestive enzymes for insect reproduction and in premises that these enzymes may affect positively or negatively the parasite development and insect vector competence, the expression of two digestive enzymes, chymotrypsin-like serine protease and carboxypeptidase were evaluated.

**Figure 3:**
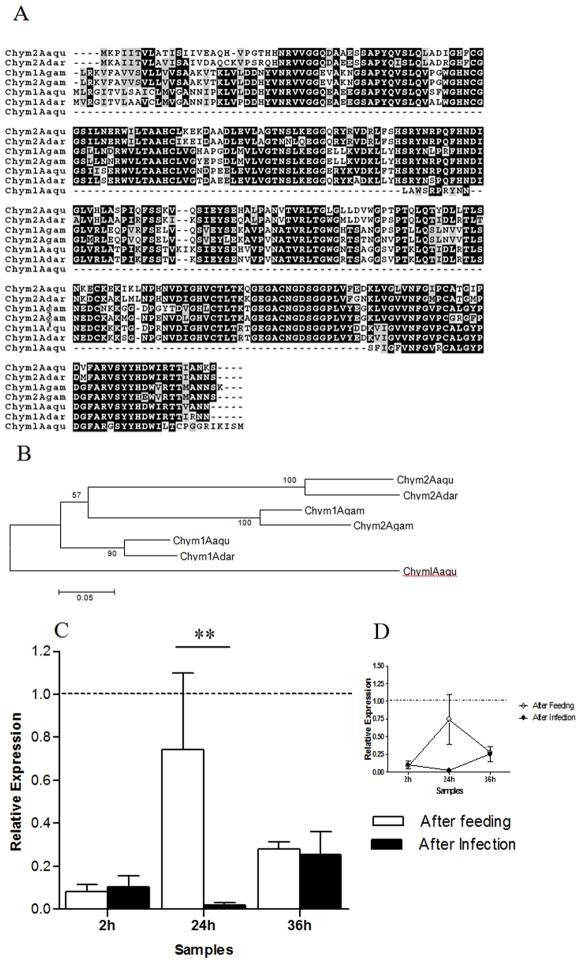
Characterization of chymotrypsin-like protease (Chymlp) induction in mosquitoes fed on sugar, blood, or infected blood. A: Multiple aminoacid sequence alignment of mosquito Chymlp sequences. B: Phylogenetic tree constructed using neighbor-joining method. C–D: Expression levels of Chymlp determined by RTPCR n sugar-fed (dotted line), blood-fed and *P. vivax* infected females. Chym–chymotrypsin, h–hours. Accession numbers of serine proteases from: *A. aquasalis* (Aaqu) (Chyml - GR486809, Chym1 - O97097, Chym2 - O97098); *A. darlingi* (Adar) (Chym1- O97099 and Chym2 - O97100); and *A. gambiae* (Agam) (Chym1 - ENSANGP00000024897, Chym2 - ENSANGP00000026162. +−: s.e.m.; * *p*<0.05, ** 0.03>*p*>0.01, *** *p*<0.01.

Twelve serine proteases sequences were obtained through subtractions. Seven of these were found in the 2hI-F library, three in the 24hF-I library, one in the 24hI-F library and one in the 2hF-I library (Tables S1, S2, S3, S4). One chymotrypsin-like molecule (Figures 3A and B) from the 2hI-F library (GR486809) was evaluated by RTPCR. This gene was not expressed in immature stages and males. Phylogenetic analysis showed this gene to be related to digestive serine proteases, which was corroborated by up-regulated expression at 24 hours AF (Figures 3C and D). Interestingly, *P. vivax* infection decreased the expression of this gene (Figures 3C and D). In contrast, some previous work in other mosquitoes showed differences in digestive enzymes activity but not in mRNA levels after pathogen challenge [17], [18]. The modulation of chymotrypsin-like serine protease transcription by the parasite could increase its survival and development in the insect, since early insect forms of *Plasmodium* are susceptible to protease digestion [19]. Parasite interference in the signaling pathways leading to transcription could explain this observation. Recently, Brandon *et al.* [20] described that the target of rapamycin (TOR) kinase, which has been implicated in nutrient sensing, was involved in early trypsin transcription and synthesis in the mosquito *A. aegypti* midgut in response to feeding. Also, three sequences related to mosquitoes GATA transcription factor, final downstream step in the TOR pathway, were found in the 2hF-I library. In *A. gambiae* GATA has been associated with upstream sequences of the trypsin genes [21]. This pathway may also be related to the reduction of the infected insects' embryogenesis, since association between TOR pathway and vitellogenin production was described in *A. aegypti* [22].

**Figure 4:**
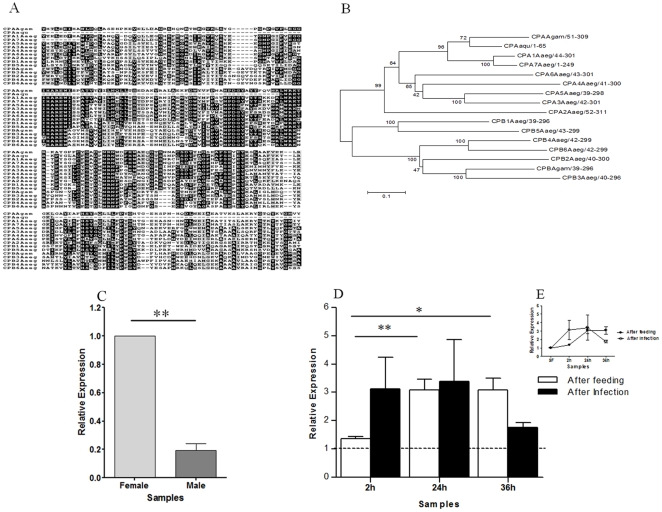
Characterization of carboxypeptidase induction in mosquitoes fed on sugar, blood, or infected blood. A: Multiple aminoacid sequence alignment of *A. aquasalis* and *A. gambiae* carboxypeptidases. B: Phylogenetic tree constructed using neighbor-joining method. C–E: Expression levels of carboxypeptidase determined by RTPCR following various feeding regimens. C: Sugar-fed males and females, D and E: sugar-fed (dotted line and SF), blood-fed (AF) and *P. vivax* infected (AI) females. h–hours. Accession numbers of carboxypeptidase sequences from: *A. aquasalis* (Aaqu) (CP–consensus of GR486815 and GR486794); *A. gambiae* (Agam) (CPA - AGAP009593 and CPB-CAF28572.1); and *A. aegypti* (CPA1-AAD47827.1, CPA2-AAT36726.1, CPA3-AAT36727.1, CPA4-AAT36728.1, CPA5-AAT36729.1, CPA6-AAT36730.1, CPA7-AAT36731.1, CPB1-AAT36735.1, CPB2-AAT36733.1, CPB3-AAT36734.1, CPB4-ABO21075.1, CPB5-ABO21076.1, and CPPB6-ABO21077.1). +−: s.e.m.; * *p*<0.05, ** 0.03>*p*>0.01, *** *p*<0.01.

Two carboxypeptidase clones representing non-overlapping regions of the same cDNA (GR486815 and GR486794) were found only in the 2hI-F library. This gene was more closely related to carboxypeptidase A than B based on phylogenetic analyses (Figures 4A and B). Expression was low in males in contrast with SF females (Figure 4C). No significant differences in mRNA levels were observed for this digestive enzyme in *A. aquasalis* AF and AI (Figures 4D and E). This is in contrast with results observed with carboxypeptidase B1 and 2 of *A. gambiae* that are up-regulated by *P. falciparum* [23], and with observations of blood meal induction of different classes of carboxypeptidase A and B of *A. aegypti* [24]. Lavazec *et al.* [2] demonstrated that antibodies against carboxypeptidase B reduced *P. falciparum* development in *A. gambiae.*

### Immunity-related genes

**Figure 5:**
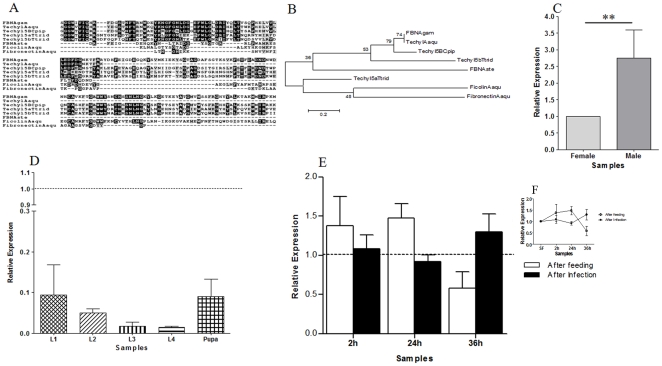
Characterization of techylectin related protein induction in mosquitoes fed on sugar, blood, or infected blood. A: Multiple aminoacid sequence alignment of mosquito fibrinogen related proteins. B: Phylogenetic tree for fibrinogen constructed based on the neighbor-joining method. C–F: Expression levels of techylectin related protein in *A. aquasalis* following different feeding regimens. C: sugar-fed males and females, D: immature stages, E and F: sugar-fed females (dotted line), and females after feeding and after *P. vivax* infection . h–hours, L1–first instar larva, L2–second instar larva, L3–third instar larva and L4–fourth instar larva. Accession numbers of fibrinogen sequences from: *A. aquasalis* (Aaqu) (Techyl - GR486377, Fibronectin - GR486898 and Ficolin - GR487133); *A. gambiae* (Agam) (FBN-AGAP004917-PA); *A. stephensi* (Aste) (FBN–CB602443), *C. pipiens* (Cpip) (Techyl5B - CPIJ000938); and *Tachypleus tridentatus* (Ttrid) (Techyl5a - AB024737 and Techyl5b - AB024738). +−: s.e.m.; * *p*<0.05, ** 0.03>*p*>0.01, *** *p*<0.01.

Although we found few immunity related genes in our subtractions, those detected should be good candidates for the development of strategies to interrupt malaria transmission and were therefore further characterized. Three fibrinogen related sequences were identified in libraries 2hF-I (techylectin-like) and 2hI-F (ficolin and fibronectin-like). The expression of the first one (GR486377) related to mosquito and horse-crab techylectins (Figures 5A and B), a molecule important in mosquito immune activation [25], was evaluated. Fibrinogen was expressed in immature stages of *A. aquasalis*, mainly in first instar larvae (L1) and pupae (Figure 5C) and the expression in males was higher than in SF females (Figure 5D). No significant expression of this techylectin related protein was seen 2, 24 and 36 hours AF and AI in females (Figures 5E and F). Nevertheless, a modest two fold increase of mRNA expression was seen 36 hours AI. Similar results were seen in other mosquito species, where levels of proteins with fibrinogen domains were increased in *A. gambiae* immediately after challenge with Gram-negative plus Gram-positive bacteria, and 24 hours after *P. berghei* infection [26]. These levels were also increased 48 hours after infection of *A. stephensi* with *P. berghei* [6]. The increase of this gene expression 36h AI can be important for early oocysts recognition and insect immune activation.

**Figure 6:**
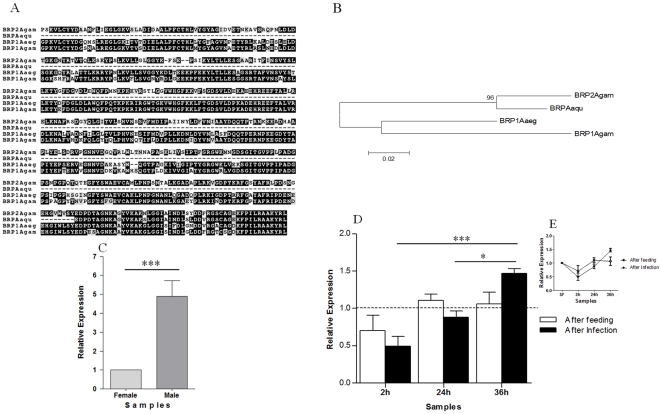
Characterization of BRP related protein induction in mosquitoes fed on sugar, blood, or infected blood. A: Multiple aminoacid sequence alignment of mosquito BRPs. B: Phylogenetic tree for BRP sequences based on the neighbor-joining method. C–E: Expression levels of BRP determined by RTPCR following various feeding regimens. C: Sugar-fed males and females, D and E: sugar-fed (dotted line and SF), blood-fed (AF) and *P. vivax* infected (AI) females. h–hours. Accession numbers of BRP sequences from: *A. aquasalis* (Aaqu) (GR486800); *A. gambiae* (Agam) (BRP1 - AGAP008061 and BRP2 - AGAP008060) and *A. aegypti* (Aaeg) (BRP1 - AAEL001965). +−: s.e.m.; * *p*<0.05, ** 0.03>*p*>0.01, *** *p*<0.01.

A sequence with 94% similarity to bacteria responsive protein (BRP) (GR486800) was identified in the 2hI-F library. Phylogenetic analysis showed the deduced peptide to be more related with BRP2 than BRP1 of *A. gambiae* (Figures 6A and B). BRP proteins are modulated by bacterial and peptidoglycan challenges in *A. gambiae* [27]. BRP2 presented higher expression in males than SF females (Figure 6C) and was induced by *P. vivax* infection. The induction of BRP2 by *P. vivax* 24 and 36 hours AI (Figures 6D and E) indicates that the parasite passage and persistence in the basal lamina of the midgut could be stimulating the immune system. According to Shi and Paskewitz [27], BRP proteins may have a function in cell proliferation or regulation of immune cell migration and aggregation. Then, increased levels of BRP2 production in *A. aquasalis* may promote these secondary immune functions in haemolymph, in response to *P. vivax* presence.

**Figure 7:**
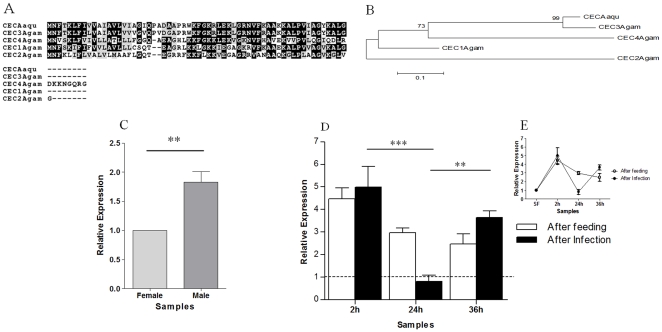
Characterization of cecropin induction in mosquitoes fed on sugar, blood, or infected blood. A: Multiple aminoacid sequence alignment of *A. aquasalis* and *A. gambiae* cecropin sequences. B: Phylogenetic tree constructed using neighbor-joining method. C–E: Expression levels of cecropin determined by RTPCR following various feeding regimens. C: Sugar-fed males and females, D and E: sugar-fed (dotted line and SF), blood-fed (AF) and *P. vivax* infected (AI) females. h–hours. Acession numbers of cecropin (cec) sequences from: *A. aquasalis* (Aaqu) (consensus of GR486610 and GR486612), and from *A. gambiae* (Agam) (Cec1 - AGAP000693-PA, Cec2 - AGAP000692-PA, Cec3–AGAP000694-PA and Cec4 - AGAP006722-PA). +−: s.e.m.; * *p*<0.05, ** 0.03>*p*>0.01, *** *p*<0.01.

Cecropin is a potent AMP with a wide range of antimicrobial targets. Two overlapping ESTs (GR486610 and GR486612) with high similarity (96%) to cecropin were found in the 2hF-I library. These two ESTs generated the complete open reading frame for an *A. aquasalis* cecropin protein (Fig. 7A). Phylogenetic analyses showed that this cecropin was closely related with cecropin 3 (or B) of *A. gambiae* (Figures 7A and B). Males presented more mRNA for cecropin than SF females (Figure 7C). In females, cecropin was up-regulated 2 hours after ingestion of both infected blood or uninfected blood and decreased at 24 and 36 hrs post blood feeding (Figures 7D and E). This induction may be a strategy to control the commensal bacterial burst induced by blood nutrients [28] due the negative regulation of reactive oxygen species production in response to heme toxicity [29]. Still, in *A. aquasalis* this gene was down-regulated 24 hours AI (Figures 7D and E), at a time when *Plasmodium* are passing through the mosquito midgut, strongly activating the immune system and producing AMPs in other mosquito species [30]. This cecropin down-regulation in *A. aquasalis* upon *P. vivax* infection is in contrast with findings in *A. aegypti* and *A. gambiae* infected with bacteria, filamentous fungi, yeast and *Plasmodium*, which cause an increase in AMP production [31]-[32]. *A. gambiae* transgenic mosquitoes overexpressing cecropin reduced *P. berghei* infection by 60% [33]. These observations suggest that the infection with *P. vivax* could be regulating the bacteria load in the insect or suppressing some signaling pathway, as NF-κB pathway, that leads to the increase of this AMP. The down-regulation of cecropin in *A. aquasalis* infected by *P. vivax* is probably important for parasite survival and development in the vector.

**Figure 8:**
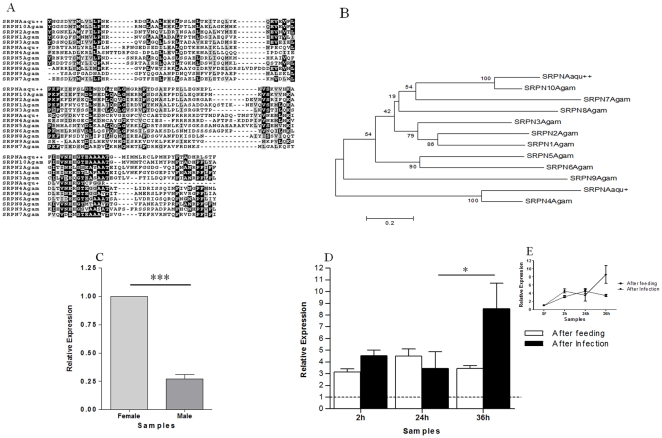
Characterization of serpin induction in mosquitoes fed on sugar, blood, or infected blood. A: Multiple aminoacid sequence alignment of *A. aquasalis* and *A. gambiae* serpin sequences. B: Phylogenetic tree constructed using neighbor-joining method. C–E: Expression levels of serpin determined by RTPCR following various feeding regimens. C: Sugar-fed males and females, D and E: sugar-fed (dotted line and SF), blood-fed (AF) and *P. vivax* infected (AI) females. A: Multiple aminoacid sequence alignment of *A. aquasalis* and *A. gambiae* serpin sequences. B: Phylogenetic tree was performed using neighbor-joining method. C - E: Relative expression of serpin in sugar-fed males and females (C); sugar-fed (dotted line), blood-fed and *P. vivax* infected females (D - E). h–hours. Accession numbers of serpin (SRPN) sequences from: *A. gambiae* (Agam) (SRPN1–AGAP006909-PA, SRPN2–AGAP006911-PA, SRPN3 - AGAP006910-PA, SRPN4–AGA009679-PA, SRPN5–AGAP009221-PA, SRPN6–AGA009212-PA, SRPN7AGAP007693-PA, SRPN8–AGAP003194-PA, SRPN9–AGAP003139-PA, and SRPN10–AJ420785). + Sequence of serpin found in the 2hAF-I library (GR486572). ++ *A. aquasalis* serpin sequence from GenBank, accession number EX809758. +−: s.e.m.; * *p*<0.05, ** 0.03>*p*>0.01, *** *p*<0.01.

Serpins play a critical role in the regulation of invertebrate innate immune responses [34] and some serpins were shown to be important for the successful development of *Plasmodium* in the insect [e.g. 35]. A unique EST related to serpin was found in the 2hF-I library (GR486572). Blast results and phylogenetic approaches showed the *A. aquasalis* serpin to group with inhibitory proteins more related to the *A. gambiae* serpin 4 (Figures 8A and B), which, in this mosquito, was induced by *Staphylococcus aureus* and *Escherichia coli*, but not by *Plasmodium* infection [36]. In contrast, in *A. aquasalis*, the expression of this gene was higher 36 hours after *P. vivax* infection (Figures 8D and E). Expression was also higher in SF females than in males (Figure 8C). Our results indicate that increased expression of this serpin in *A. aquasalis* may be triggered by parasite passage through the midgut epithelium and the parasite exposure in the haemolymph, and that this increase can be responsible for infection success of *P. vivax* by the suppression of the mosquito immune response such as melanization. Functional studies need to be done to prove if this serpin acts as a negative immunomodulator. If *A. aquasalis* serpin indeed carries out a protective function for the parasite, this protein would be an ideal target for an anti-malaria strategy.

### General comments and conclusions

RTPCR experiments revealed high expression of mRNAs for some digestion and immune genes in sugar-fed female and male *A. aquasalis*. Males presented more elevated levels of mRNA for immunity genes (BRP and cecropin) and lower levels of the negative immune regulator serpin than females, indicating that these insects are immunologically ready for the ingestion of contaminated sugar or other fluids. On the other hand, prior to blood feeding, females presented higher levels of mRNA for the digestive enzymes chymotrypsin-like serine protease and carboxypeptidase, possibly involved with digestion of blood necessary for egg maturation, than males.

Although blood was obtained from many different donors, and RNA was extracted from pools of insects, we cannot totally exclude the possibility, albeit improbable, that some of the gene expression differences observed were due to heterogeneity of the blood ingested by the insects or to anemic state commonly found in malaria patients [37].

Our present studies revealed new aspects of interactions between the important American vector *A. aquasalis* and its natural parasite *P. vivax*. Subtraction experiments and RTPCR showed that the early steps of *P. vivax* infection did not activate the *A. aquasalis* immune system as powerfully as observed in other mosquito-*Plasmodium* pairs. In the case of *A. aquasalis*, the presence of the parasite in insect haemolymph 36 hours after infection, rather than its presence in the midgut or during passage through its epithelium 24 hours after infection, appeared to correlate with the induction of a potent anti-microbial immune response. This information could contribute for the development of new strategies intended to interrupt the *P. vivax* cycle within its vector. Differences detected in relation to others malaria vectors might help direct new malaria transmission-blocking strategies specific for *A. aquasalis*. Garver *et al.* [38] showed differences of immune response of *A. gambiae* to *P. falciparum* and *P. berghei,* and also revealed some common aspects of the immune response of three anopheline species, *A. gambiae, A. stephensi* and *Anopheles albimanus* to a single *Plasmodium* species, *P. falciparum*. These observations demonstrate the importance of obtaining specific information on the various malaria parasite and vector pairs, since it apparently is impossible to predict their responses and outcome of infection.

## Materials and Methods

### Ethics statement

For the acquisition of *P. vivax* infected human blood, eight patients were selected among the people visiting the Health Center (Posto Estadual de Saúde da Vigilância em Saúde do Município de Iranduba, Distrito de Cacau Pirêra, Amazonas, Brazil) looking for malaria diagnosis and treatment during outbreaks. Diagnosis was performed by Giemsa stained blood smear. After positive diagnosis and visualization of gametocytes, patients were interviewed and inquired about the possibility of volunteer donation of a small amount of blood for research purposes. After verbal agreement, a term of consent was first read to the potential volunteers, with detailed verbal explanation, and, after final consent, signed by the patient. After this, one 200 µl sample of venous blood was drawn from each patient and placed in heparinized tubes. Blood samples were kept under refrigeration in an ice box (at approximately 15°C) for about 15 minutes, taken to the laboratory and used to feed *A. aquasalis*. Patient selection criteria were: to be *P. vivax* positive, to have about 4–8% of circulating gametocytes, determined by the National Institutes of Health international protocols, and to consent to be part of the research (consent form was approved by the Brazilian Ministry of Health, National Council of Health, National Committee of Ethics in Research (CONEP), written approval number 3726).

### Insects and infection

*A. aquasalis* were reared at 27°C and 80% humidity. Insect infections were performed in an endemic area of Manaus, Amazonas state. Malaria patients were diagnosed by microscopic examination of Giemsa-stained blood smears. Infected or control blood was offered to the insects by artificial feeding at 37°C constant temperature, maintained using a water circulation system, to prevent exflagellation of microgametocytes. After the experimental feeding, the mosquitoes were kept in cages and given 20% sucrose *ad libitum*. Whole mosquitoes were separated in pools of 25 insects for subtraction experiments at 2 and 24 hours AF and AI, and at 2, 24 and 36 hours AF and AI were separated in pools of 5 insects for RTPCR. Infection was evaluated by PCR using a specific *Plasmodium* 18 s rRNA gene [39].

### Subtraction experiments

RNA from *A. aquasalis* fed on human blood infected or not with *P. vivax* was extracted with TRIzol (Invitrogen), messenger RNA (mRNA) was purified using the NucleoTrap® mRNA Mini Purification Kit (Clontech) and cDNAs used for subtractions were synthesized using the PCR-Select™ cDNA Subtraction Kit (Clontech). Equal amounts of cDNA from each sample were pooled to construct the four libraries using cDNAs from 2 and 24 hours infected minus non-infected insects and 2 and 24 hours non-infected minus infected insects. After two PCR rounds, the amplicons were cloned into pGEM®-T Easy Vector (Promega) and utilized to transform high efficiency DH5-α *E. coli*. Sequencing of 2,138 selected clones was performed using an ABI 3700 sequencer (Applied Biosystems) in the PDTIS/FIOCRUZ Sequencing Platform.

### Sequences annotation

The sequences obtained were submitted to the STINGRAY platform (System for Integrated Genomic Resources and Analyses) (http://stingray.biowebdb.org/). Vector and primer sequences were trimmed and quality evaluated by Phred, Phrap and Consed programs. The sequences were clustered using the CAP3 program and clusters were searched for similarity using the BLASTN and BLASTX algorithm with sequences of insects, plasmodia and different databases (RefSeq, UniRef, UniProt, InterProt, KOG, COG, Prk, Smart and CDD). The cutoff e-values utilized were ≥10^-5^ for tBLASTX similarity and 10^-10^ for BLASTN. Sequences were annotated and grouped in functions. Nucleotide sequences have been submitted to Genbank and their respective accession numbers are indicated in Tables S1, S2, S3, S4.

### RTPCR

RNA was extracted from whole insects submitted to different experimental conditions. The extracted RNA was treated with RQ1 DNAse free-RNAse (Promega) and utilized for cDNA synthesis. RTPCR reactions were performed using the SyberGreen fluorescent probe in an ABI 7000 machine (Applied Biosystems). The PCR cycles used were 50°C 2 min, 95°C 10 min, 95°C 15 sec and 63°C 1 min for 35 times for all reactions. Primer sequences and amplicon lengths are listed in Table S5. The relative expression of the selected genes was based on gene expression CT difference formula [40]. Quantifications were normalized in relation to the housekeeping gene rp49 [41]. All the experiments were performed using four to six biological replicates. The statistic method used in the analysis was ANOVA test with multiple comparisons of Tukey or Games-Howell. When the parametric model (ANOVA) was not adequate, we utilized the Kruskal-Wallis test with multiple comparisons of Mann-Whitney. For the male versus female analyses the t-student or the Wilcoxon tests were utilized. All tests were performed with reliable level of 95% (α = 0.05). The statistical analyses were accomplished using the *Graph pad Prism5*®, R, software.

### Sequence analyses

The sequences were aligned using the ClustalW (http://www.ebi.ac.uk/Tools/clustalw2/) and presented with BOXSHADE (http://bioweb.pasteur.fr/seqanal/interfaces/boxshade.html) programs. For phylogenetic analyses, the sequence alignments were examined with the Mega program (Molecular Evolutionary Genetics analysis, version 4) [42]. Relationships between the sequences were assessed by neighbor-joining method with amino acid distances with 1,000 replications in the bootstrap test.

## Supporting Information

Figure S1 PCR to confirm A. aquasalis experimental infection with P. vivax. MW - molecular weight marker, Pv18s - P. vivax 18 s rRNA gene, I - infected insects, C- - negative control, C+ - blood of humans infected with P. vivax, I (25) - pool of 25 P. vivax infected insects and I (1) - one P. vivax infected insect.
(0.69 MB TIF)

Figure S2 Differentially expressed products amplified after different subtractions (2hF-I 2hI-F, 24hF-I and 24hI-F). W - molecular weight marker, F-I - cDNA after feeding minus after infection and I-F - cDNA after infection minus after feeding. h - Hours of feeding or infection. bp - base pairs.
(0.82 MB TIF)

Table S1 List of sequences from the 2 hours non-infected minus infected insects library. Sequences with significant similarity on BLASTN or BLASTX were grouped based on the function of the homologous protein.
(0.29 MB DOC)

Table S2 List of sequences from the 2 hours infected minus non-infected insects library. Sequences with significant similarity on BLASTN or BLASTX were grouped based on the function of the homologous protein.
(0.28 MB DOC)

Table S3 List of sequences from the 24 hours non-infected minus infected insects library. Sequences with significant similarity on BLASTN or BLASTX were grouped based on the function of the homologous protein.
(0.13 MB DOC)

Table S4 List of sequences from the 24 hours infected minus non-infected insects library. Sequences with significant similarity on BLASTN or BLASTX were grouped based on the function of the homologous protein.Sequences with significant similarity on BLASTN or BLASTX were grouped based on the function of the homologous protein.
(0.12 MB DOC)

Table S5 Primers used for quantitative PCR.
(0.03 MB DOC)
