# Supplementary figures and images for: Anopheles aquasalis Infected by Plasmodium vivax Displays Unique Gene Expression Profiles when Compared to Other Malaria Vectors and Plasmodia
Source: PLoS One. 2010 Mar 22;5(3):e9795. doi: 10.1371/journal.pone.0009795 (PMC2842430; doi:10.1371/journal.pone.0009795)

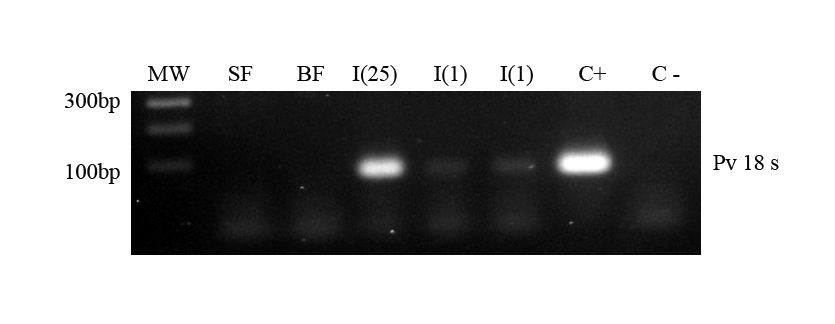

Supplement: Figure S1 — PCR to confirm A. aquasalis experimental infection with P. vivax. MW - molecular weight marker, Pv18s - P. vivax 18 s rRNA gene, I - infected insects, C- - negative control, C+ - blood of humans infected with P. vivax, I (25) - pool of 25 P. vivax infected insects and I (1) - one P. vivax infected insect. (0.69 MB TIF) [file pone.0009795.s001.tif]

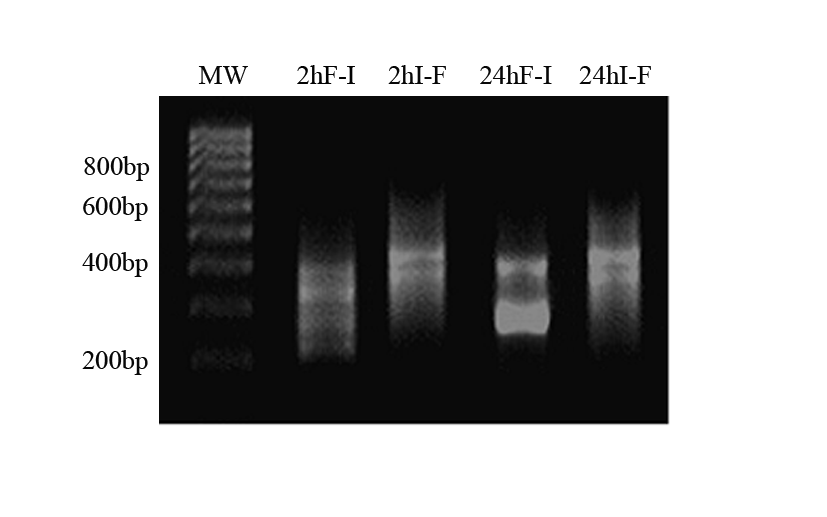

Supplement: Figure S2 — Differentially expressed products amplified after different subtractions (2hF-I 2hI-F, 24hF-I and 24hI-F). W - molecular weight marker, F-I - cDNA after feeding minus after infection and I-F - cDNA after infection minus after feeding. h - Hours of feeding or infection. bp - base pairs. (0.82 MB TIF) [file pone.0009795.s002.tif]
