# Supplementary material for: Anopheles aquasalis Infected by Plasmodium vivax Displays Unique Gene Expression Profiles when Compared to Other Malaria Vectors and Plasmodia
Source: PLoS One. 2010 Mar 22;5(3):e9795. doi: 10.1371/journal.pone.0009795 (PMC2842430; doi:10.1371/journal.pone.0009795)
Supplement: Table S1 — List of sequences from the 2 hours non-infected minus infected insects library. Sequences with significant similarity on BLASTN or BLASTX were grouped based on the function of the homologous protein. (0.29 MB DOC) [file pone.0009795.s003.doc]

| **Accession number** | | **Number of reads** | **G+C Content** | **CDS Length** | **Annotated Description** | **E-value** | **Score** | **Organism/Database** | **Gene**  **Accession**  **no.** |
| --- | --- | --- | --- | --- | --- | --- | --- | --- | --- |
| **Signal transduction mechanism** | | | | | | | | | |
| GR486343 | | 1 | 59% | 87 | Rhodopsin receptor 3 | 1.0e-08 | 54 | Anopheles_gambiae.AgamP3.50.pep.all.fa | AGAP001178-PA |
| GR486481 | | 1 | 46% | 243 | 14-3-3 protein | 6.0e-41 | 163 | Anopheles_gambiae.AgamP3.50.pep.all.fa | AGAP007643-PB |
| GR486499 | | 1 | 57% | 63 | Phosrestin i (arrestin b or 2) | 2.0e-06 | 47 | Aedes_aegypti.AaegL1.50.pep.all.fa | AAEL003116-PA |
| **Biomolecules degradation** | | | | | | | | | |
| GR486394 | 1 | | 54% | 93 | Neprilysin | 8.0e-14 | 71 | Anopheles_gambiae.AgamP3.50.pep.all.fa | AGAP001791-PA |
| GR486445 | 1 | | 51% | 132 | Proteasome activator subunit | 2.0e-19 | 89 | Anopheles_gambiae.AgamP3.50.pep.all.fa | AGAP000308-PA |
| GR486421 | 1 | | 58% | 462 | Arginine methyltransferase-interacting protein | 1.0e-74 | 275 | Anopheles_gambiae.AgamP3.50.pep.all.fa | AGAP008075-PA |
| GR486410 | 2 | | 58% | 177 | Metalloprotease | 1.0e-27 | 116 | Anopheles_gambiae.AgamP3.50.pep.all.fa | AGAP000935-PA |
| GR486444 | 1 | | 56% | 330 | Arginine methyltransferase-interacting protein | 4.0e-66 | 246 | Anopheles_gambiae.AgamP3.50.pep.all.fa | AGAP008075-PA |
| GR486476 | 1 | | 56% | 330 | E3 ubiquitin ligase interacting with arginine methyltransferase | 5.0e-66 | 246 | Anopheles_gambiae.AgamP3.50.pep.all.fa | AGAP008075-PA |
| GR486472 | 2 | | 52% | 222 | Lipase 1 | 7.0e-20 | 91 | Anopheles_gambiae.AgamP3.50.pep.all.fa | AGAP002353-PA |
| GR486479 | 1 | | 47% | 210 | Ubiquitin specific protease family C19-related | 1.0e-34 | 140 | Anopheles_gambiae.AgamP3.50.pep.all.fa | AGAP006652-PA |
| GR486446 | 3 | | 53% | 417 | Alpha-amylase | 4.0e-51 | 196 | Aedes_aegypti.AaegL1.50.pep.all.fa | AAEL000667-PA |
| GR486569 | 1 | | 53% | 339 | Dipeptidyl peptidase 4 | 3.0e-58 | 219 | Anopheles_gambiae.AgamP3.50.pep.all.fa | AGAP008176-PA |
| GR486703 | 1 | | 56% | 180 | Acid phosphatase | 2.0e-18 | 86 | Anopheles_gambiae.AgamP3.50.pep.all.fa | AGAP007400-PA |
| GR486555 | 1 | | 49% | 147 | Serine protease | 7.0e-34 | 129 | anopheles_aquasalis_EST.fasta | EX809821 |
| **Replication, translation and transcription** | | | | | | | | | |
| GR486346 | 1 | | 44% | 84 | 28S large subunit ribosomal RNA | 7.0e-15 | 70 | anopheles_darlingi_nucleotideo.fasta | AF417805 |
| GR486357 | 1 | | 43% | 87 | 28S large subunit ribosomal RNA | 3.0e-14 | 68 | anopheles_darlingi_nucleotideo.fasta | AF417805 |
| GR486366 | 1 | | 56% | 288 | ATP-binding cassette, sub-family E (OABP), member 1 | 1.0e-47 | 183 | Aedes_aegypti.AaegL1.50.pep.all.fa | AAEL010059-PA |
| GR486347 | 1 | | 44% | 84 | 28S large subunit ribosomal RNA | 7.0e-15 | 70 | anopheles_darlingi_nucleotideo.fasta | AF417805 |
| GR486367 | 1 | | 43% | 87 | 28S large subunit ribosomal RNA | 3.0e-14 | 68 | anopheles_darlingi_nucleotideo.fasta | AF417805 |
| GR486332 | 3 | | 51% | 234 | 40S ribosomal protein S11 | 1.0e-40 | 153 | anopheles_darlingi_ptna.fasta | ACI30064 |
| GR486392 | 1 | | 47% | 249 | 50S/60S ribosomal protein L14/L23 | 1.0e-44 | 167 | anopheles_darlingi_ptna.fasta | ACI30146 |
| GR486337 | 1 | | 54% | 318 | 60S ribosomal protein NHP2/L7A | 8.0e-47 | 181 | Anopheles_gambiae.AgamP3.50.pep.all.fa | AGAP012204-PA |
| GR486328 | 1 | | 53% | 150 | Ubiquitin/60S ribosomal protein L40 fusion | 7.0e-10 | 57 | apis_mellifera_ptna.fa | XP_397323 |
| GR486344 | 1 | | 47% | 129 | 60S ribosomal protein L14/L17/L23 | 8.0e-22 | 91 | anopheles_darlingi_ptna.fasta | ACI30146 |
| GR486409 | 2 | | 52% | 267 | NEFA-interacting nuclear protein NIP30 | 2.0e-18 | 86 | Anopheles_gambiae.AgamP3.50.pep.all.fa | AGAP010768-PA |
| GR486464 | 2 | | 54% | 129 | Histone acetyltransferase gcn5 | 2.0e-17 | 83 | Anopheles_gambiae.AgamP3.50.pep.all.fa | AGAP004434-PA |
| GR486490, GR486416 | 3 | | 53% | 1179 | DEAD box ATP-dependent RNA helicase | 1.0e-167 | 583 | Anopheles_gambiae.AgamP3.50.pep.all.fa | AGAP008578-PA |
| GR486423 | 2 | | 56% | 621 | Transient receptor potential channel 4 | 2.0e-83 | 304 | Anopheles_gambiae.AgamP3.50.pep.all.fa | AGAP010630-PA |
| GR486452 | 3 | | 54% | 285 | Mago nashi protein | 6.0e-49 | 187 | Anopheles_gambiae.AgamP3.50.pep.all.fa | AGAP010755-PA |
| GR486469 | 1 | | 53% | 150 | Proliferation-associated 2g4 | 2.0e-19 | 90 | Aedes_aegypti.AaegL1.50.pep.all.fa | AAEL012312-PA |
| GR486467 | 1 | | 53% | 285 | Endoribonuclease XendoU | 1.0e-46 | 181 | Anopheles_gambiae.AgamP3.50.pep.all.fa | AGAP002925-PA |
| GR486448 | 1 | | 53% | 291 | U4/U6-associated splicing factor PRP4 |  |  |  |  |
| GR486487 | 1 | | 59% | 114 | Ribosomal protein S15p/S13e | 3.0e-15 | 75 | Anopheles_gambiae.AgamP3.50.pep.all.fa | AGAP005947-PA |
| GR486580 | 1 | | 49% | 129 | 60S ribosomal protein L14/L17/L23 | 5.0e-21 | 88 | anopheles_darlingi_ptna.fasta | ACI30146 |
| GR486621 | 1 | | 54% | 177 | 40S ribosomal protein S11 | 2.0e-39 | 116 | anopheles_darlingi_ptna.fasta | ACI30064 |
| GR486558 | 1 | | 56% | 135 | Transcriptional Coactivator p15 (PC4) | 3.0e-14 | 73 | Aedes_aegypti.AaegL1.50.pep.all.fa | AAEL008736-PB |
| GR486591 | 1 | | 54% | 204 | 40S ribosomal protein S7 | 2.0e-31 | 129 | Anopheles_gambiae.AgamP3.50.pep.all.fa | AGAP010592-PA |
| GR486601 | 1 | | 59% | 141 | 40S ribosomal protein S4 | 2.0e-24 | 99 | anopheles_darlingi_ptna.fasta | ACI30066 |
| GR486576 | 2 | | 49% | 291 | Nonsense-mediated mRNA decay 2 protein |  |  |  |  |
| GR486699 | 1 | | 62% | 117 | Transcription factor GATAa2 | 2.0e-07 | 50 | cpipiens.PEPTIDES-CpipJ1.1.fa | CPIJ008350-PA |
| GR486701 | 1 | | 47% | 276 | 60S ribosomal protein L14/L17/L23 | 6.0e-35 | 134 | anopheles_darlingi_ptna.fasta | ACI30146 |
| GR486632 | 1 | | 54% | 225 | Ribosomal protein L28 | 2.0e-15 | 78 | Anopheles_gambiae.AgamP3.50.pep.all.fa | AGAP008376-PA |
| GR486641 | 1 | | 60% | 141 | Transcription factor GATA-4 | 7.0e-10 | 58 | Aedes_aegypti.AaegL1.50.pep.all.fa | AAEL010222-PB |
| GR486542 | 1 | | 63% | 117 | Transcription factor GATA | 1.0e-08 | 54 | cpipiens.PEPTIDES-CpipJ1.1.fa | CPIJ008350-PA |
| GR486604 | 1 | | 57% | 228 | 60S ribosomal protein L29 | 3.0e-41 | 155 | anopheles_darlingi_ptna.fasta | ACI30089 |
| GR486570 | 1 | | 58% | 69 | Translationally-controlled tumor protein | 4.0e-08 | 52 | Anopheles_gambiae.AgamP3.50.pep.all.fa | AGAP002667-PA |
| **Metabolism** | | | | | | | | | |
| GR486454 | 1 | | 60% | 174 | Aspartate ammonia lyase | 1.0e-33 | 144 | cpipiens.SUPERCONTIGS-Johannesburg.CpipJ1.fa | DS231997.1 |
| GR486439 | 1 | | 61% | 144 | Glycogenin | 8.0e-17 | 83 | cpipiens.PEPTIDES-CpipJ1.1.fa | CPIJ013596-PA |
| GR486511 | 1 | | 51% | 615 | Proline oxidase | 8.0e-96 | 345 | cpipiens.PEPTIDES-CpipJ1.1.fa | CPIJ009461-PA |
| GR486431 | 2 | | 52% | 255 | Alanine aminotransferase | 7.0e-42 | 164 | Anopheles_gambiae.AgamP3.50.pep.all.fa | AGAP000901-PA |
| GR486459 | 3 | | 56% | 279 | Threonine dehydrogenase | 2.0e-51 | 195 | Anopheles_gambiae.AgamP3.50.pep.all.fa | AGAP011948-PA |
| GR486681 | 2 | | 52% | 354 | Choline/ethanolamine kinase | 3.0e-29 | 118 | Anopheles_gambiae.AgamP3.50.pep.all.fa | AGAP007957-PA |
| GR486674 | 1 | | 54% | 126 | Farnesoic acid O-methyltransferase-like protein | 1.0e-16 | 80 | Anopheles_gambiae.AgamP3.50.pep.all.fa | AGAP006103-PA |
| GR486638 | 1 | | 51% | 336 | Choline/ethanolamine kinase | 1.0e-22 | 100 | Anopheles_gambiae.AgamP3.50.pep.all.fa | AGAP007957-PA |
| GR486566 | 2 | | 53% | 201 | HAD-superfamily hydrolase | 3.0e-17 | 83 | Aedes_aegypti.AaegL1.50.pep.all.fa | AAEL007703-PA |
| **Defense and detoxification** | | | | | | | | | |
| GR486377 | 1 | | 45% | 207 | Fibrinogen (techylectin-5B) | 3.0e-33 | 135 | Anopheles_gambiae.AgamP3.50.pep.all.fa | AGAP004917-PA |
| GR486417 | 1 | | 56% | 192 | V-type ATP synthase beta chain | 1.0e-32 | 133 | Aedes_aegypti.AaegL1.50.pep.all.fa | AAEL005798-PA |
| GR486477 | 1 | | 54% | 213 | Integral transmembrane protein 2-related with immunoglobulin domain | 1.0e-29 | 125 | Anopheles_gambiae.AgamP3.50.pep.all.fa | AGAP009156-PA |
| GR486461 | 2 | | 53% | 465 | Apoptosis inhibitory protein 5 (API5) | 3.0e-51 | 196 | Anopheles_gambiae.AgamP3.50.pep.all.fa | AGAP009645-PA |
| GR486572 | 2 | | 53% | 369 | Serine protease inhibitor | 1.0e-23 | 103 | Anopheles_gambiae.AgamP3.50.pep.all.fa | AGAP009670-PB |
| GR486610 | 1 | | 54% | 180 | Antimicrobial peptide cecropin | 2.0e-22 | 92 | anopheles_darlingi_ptna.fasta | ACI30166 |
| GR486626 | 1 | | 50% | 210 | Internalin A | 5.0e-29 | 121 | Anopheles_gambiae.AgamP3.50.pep.all.fa | AGAP004458-PA |
| GR486612 | 2 | | 54% | 180 | Antimicrobial peptide cecropin | 2.0e-22 | 92 | anopheles_darlingi_ptna.fasta | ACI30166 |
| **Structural genes** | | | | | | | | | |
| GR486532 | 1 | | 57% | 507 | Myosin heavy chain | 3.0e-33 | 137 | Anopheles_gambiae.AgamP3.50.pep.all.fa | AGAP007523-PB |
| GR486523, | 22 | | 58% | 549 | Myosin | 2.0e-33 | 137 | Anopheles_gambiae.AgamP3.50.pep.all.fa | AGAP007523-PB |
| GR486397 | 1 | | 58% | 372 | Myosin light chain 1 | 3.0e-62 | 234 | Anopheles_gambiae.AgamP3.50.pep.all.fa | AGAP007806-PA |
| GR486468 | 1 | | 58% | 372 | Myosin light chain 1 | 3.0e-62 | 234 | Anopheles_gambiae.AgamP3.50.pep.all.fa | AGAP007806-PA |
| GR486530 | 1 | | 56% | 252 | Zeelin1 | 8.0e-32 | 132 | Anopheles_gambiae.AgamP3.50.pep.all.fa | AGAP004161-PA |
| GR486552 | 1 | | 51% | 153 | Brain chitinase and chia | 2.0e-12 | 66 | Aedes_aegypti.AaegL1.50.pep.all.fa | AAEL012467-PA |
| GR486516 | 1 | | 52% | 162 | Thymosin | 2.0e-10 | 68 | uniprot_trembl.fasta | 8343_uniprot_trembl. |
| GR486496 | 1 | | 52% | 162 | Thymosin | 2.0e-10 | 68 | uniprot_trembl.fasta | 8343_uniprot_trembl. |
| GR486492 | 1 | | 52% | 162 | Thymosin | 2.0e-10 | 68 | uniprot_trembl.fasta | 8343_uniprot_trembl. |
| GR486549 | 1 | | 59% | 105 | Gelsolin | 2.0e-11 | 63 | cpipiens.PEPTIDES-CpipJ1.1.fa | CPIJ004628-PA |
| GR486683 | 1 | | 50% | 456 | CLIP-associating protein | 8.0e-47 | 181 | Anopheles_gambiae.AgamP3.50.pep.all.fa | AGAP007623-PA |
| GR486603 | 1 | | 59% | 177 | Mucin-like peritrophin | 2.0e-32 | 125 | anopheles_darlingi_ptna.fasta | ACI30179 |
| GR486538 | 1 | | 57% | 204 | Myosin light chain 1 | 2.0e-29 | 122 | Anopheles_gambiae.AgamP3.50.pep.all.fa | AGAP001569-PA |
| **Energy metabolism** | | | | | | | | | |
| GR486534 | 4 | | 58% | 402 | AMP dependent coa ligase | 7.0e-52 | 199 | Anopheles_gambiae.AgamP3.50.pep.all.fa | AGAP008557-PA |
| GR486462 | 1 | | 61% | 393 | Dihydrolipoamide succinyltransferase | 1.0e-38 | 154 | Anopheles_gambiae.AgamP3.50.pep.all.fa | AGAP004055-PA |
| GR486403, GR486413 | 7 | | 61% | 408 | Dihydrolipoamide succinyltransferase | 1.0e-41 | 164 | Anopheles_gambiae.AgamP3.50.pep.all.fa | AGAP004055-PA |
| GR486418, GR486414 | 6 | | 59% | 399 | AMP dependent coa ligase | 4.0e-51 | 197 | Anopheles_gambiae.AgamP3.50.pep.all.fa | AGAP008557-PA |
| GR486435 | 1 | | 55% | 267 | Phosphoenolpyruvate carboxykinase | 3.0e-46 | 178 | Anopheles_gambiae.AgamP3.50.pep.all.fa | AGAP003350-PA |
| GR486453 | 1 | | 57% | 396 | Dihydrolipoamide succinyltransferase | 3.0e-25 | 110 | Anopheles_gambiae.AgamP3.50.pep.all.fa | AGAP004055-PA |
| GR486498 | 1 | | 57% | 174 | Pyruvate/2-oxoglutarate dehydrogenase complex | 3.0e-21 | 96 | Anopheles_gambiae.AgamP3.50.pep.all.fa | AGAP003136-PA |
| GR486597 | 1 | | 58% | 207 | Glutaryl-CoA dehydrogenase | 8.0e-33 | 134 | Anopheles_gambiae.AgamP3.50.pep.all.fa | AGAP008501-PA |
| **Embryogenesis** | | | | | | | | | |
| GR486396 | 1 | | 55% | 189 | Apolipophorins / vitellogenin | 3.0e-25 | 108 | Anopheles_gambiae.AgamP3.50.pep.all.fa | AGAP001826-PA |
| **Transport and secretion** | | | | | | | | | |
| GR486335 | 2 | | 50% | 270 | Calmodulin and related proteins | 5.0e-26 | 111 | Anopheles_gambiae.AgamP3.50.pep.all.fa | AGAP006182-PA |
| GR486503 | 1 | | 59% | 687 | Monocarboxylate transporter | 6.0e-87 | 315 | Anopheles_gambiae.AgamP3.50.pep.all.fa | AGAP002587-PA |
| GR486527 | 1 | | 55% | 708 | Coatomer | 1.0e-118 | 421 | Aedes_aegypti.AaegL1.50.pep.all.fa | AAEL011650-PA |
| GR486429 | 2 | | 49% | 96 | Clathrin coat assembly protein AP17 | 3.0e-11 | 64 | Anopheles_gambiae.AgamP3.50.pep.all.fa | AGAP001703-PA |
| GR486457 | 1 | | 42% | 177 | Vacuolar sorting protein PEP3/VPS18 | 4.0e-26 | 111 | Anopheles_gambiae.AgamP3.50.pep.all.fa | AGAP000983-PA |
| GR486535 | 1 | | 54% | 129 | Antigen 5-related salivary protein | 1.0e-21 | 90 | anopheles_darlingi_ptna.fasta | AAQ17073 |
| GR486671 | 1 | | 55% | 198 | Translocon-associated protein alpha | 7.0e-29 | 120 | Anopheles_gambiae.AgamP3.50.pep.all.fa | AGAP001721-PA |
| GR486702 | 1 | | 57% | 291 | Calcium-transporting atpase sarcoplasmic/endoplasmic reticulum type (calcium pump) | 2.0e-52 | 199 | Anopheles_gambiae.AgamP3.50.pep.all.fa | AGAP006186-PD |
| **Unknown protein** | | | | | | | | | |
| GR486339 | 1 | | 53% | 171 | Unknown protein |  |  |  |  |
| GR486386 | 1 | | 37% | 459 | Unknown protein |  |  |  |  |
| GR486334 | 3 | | 47% | 633 | Unknown protein with coiled-coil domain |  |  |  |  |
| GR486331 | 1 | | 44% | 624 | Unknown protein |  |  |  |  |
| GR486342 | 1 | | 45% | 87 | Unknown protein |  |  |  |  |
| GR486333 | 4 | | 45% | 159 | Unknown protein |  |  |  |  |
| GR486382 | 1 | | 51% | 180 | Unknown protein |  |  |  |  |
| GR486398 | 1 | | 45% | 168 | Unknown protein |  |  |  |  |
| GR486390 | 1 | | 46% | 420 | Unknown protein |  |  |  |  |
| GR486690 | 2 | | 50% | 471 | Unknown protein |  |  |  |  |
| GR486488 | 1 | | 50% | 813 | Unknown protein |  |  |  |  |
| GR486482 | 1 | | 45% | 519 | Unknown protein with 2Fe-2S ferredoxin-type iron-sulfur binding region signature |  |  |  |  |
| GR486405 | 1 | | 39% | 540 | Unknown protein |  |  |  |  |
| GR486447 | 1 | | 53% | 309 | Unknown protein |  |  |  |  |
| GR486406 | 1 | | 51% | 102 | Unknown protein |  |  |  |  |
| GR486393 | 2 | | 53% | 801 | Unknown protein |  |  |  |  |
| GR486415 | 1 | | 48% | 768 | Unknown protein |  |  |  |  |
| GR486463 | 1 | | 54% | 705 | Unknown protein |  |  |  |  |
| GR486480 | 1 | | 51% | 126 | Unknown protein |  |  |  |  |
| GR486515 | 1 | | 50% | 402 | Unknown protein |  |  |  |  |
| GR486474 | 3 | | 43% | 282 | Unknown protein |  |  |  |  |
| GR486424 | 1 | | 39% | 288 | Unknown protein |  |  |  |  |
| GR486521 | 1 | | 44% | 279 | Unknown protein |  |  |  |  |
| GR486550 | 1 | | 46% | 294 | Unknown protein |  |  |  |  |
| GR486513 | 1 | | 39% | 612 | Unknown protein |  |  |  |  |
| GR486545 | 1 | | 43% | 258 | Unknown protein |  |  |  |  |
| GR486685 | 1 | | 51% | 174 | Unknown protein |  |  |  |  |
| GR486599 | 1 | | 46% | 450 | Unknown protein with EGF-1 domain |  |  |  |  |
| GR486584 | 4 | | 44% | 489 | Unknown protein |  |  |  |  |
| GR486553 | 1 | | 47% | 216 | Unknown protein |  |  |  |  |
| GR486595 | 2 | | 45% | 384 | Unknown protein |  |  |  |  |
| GR486533 | 1 | | 61% | 135 | Unknown protein |  |  |  |  |
| GR486578 | 2 | | 33% | 372 | Unknown protein |  |  |  |  |
| GR486548 | 1 | | 49% | 261 | Unknown protein |  |  |  |  |
| GR486559 | 1 | | 56% | 162 | Unknown protein |  |  |  |  |
| GR486592 | 2 | | 48% | 162 | Unknown protein |  |  |  |  |
| GR486679 | 1 | | 40% | 402 | Unknown protein |  |  |  |  |
| GR486593 | 1 | | 36% | 174 | Unknown protein |  |  |  |  |
| GR486666 | 1 | | 50% | 195 | Unknown protein |  |  |  |  |
| GR486571 | 2 | | 54% | 171 | Unknown protein |  |  |  |  |
| GR486607 | 1 | | 39% | 282 | Unknown protein |  |  |  |  |
| GR486551, GR486588 | 2 | | 55% | 282 | Unknown protein |  |  |  |  |
| GR486573 | 1 | | 34% | 489 | Unknown protein |  |  |  |  |
| GR486547 | 1 | | 45% | 333 | Unknown protein with Kringle-like domain |  |  |  |  |
| GR486546 | 1 | | 43% | 285 | Unknown protein |  |  |  |  |
| GR486636 | 1 | | 46% | 300 | Unknown protein |  |  |  |  |
| GR486544 | 1 | | 37% | 615 | Unknown protein |  |  |  |  |
| GR486589 | 1 | | 42% | 150 | Unknown protein |  |  |  |  |
| GR486598 | 1 | | 54% | 201 | Unknown protein |  |  |  |  |
| GR486633 | 1 | | 54% | 114 | Unknown protein |  |  |  |  |
| GR486792 | 1 | | 53% | 90 | Unknown protein |  |  |  |  |
| GR486781 | 1 | | 55% | 210 | Unknown protein |  |  |  |  |
| GR486721 | 1 | | 55% | 150 | Unknown protein |  |  |  |  |
| **Unknown conserved protein** | | | | | | | | | |
| GR486340 | 1 | | 46% | 414 | Unknown conserved protein | 5.0e-28 | 119 | Anopheles_gambiae.AgamP3.50.pep.all.fa | AGAP006195-PA |
| GR486336 | 5 | | 50% | 60 | Unknown conserved protein | 7.0e-09 | 40 | anopheles_darlingi_EST.fasta | FK704551 |
| GR486374 | 1 | | 40% | 267 | Unknown conserved protein | 7.0e-26 | 82 | A.stephensi_EST.fasta | EX225359 |
| GR486341 | 1 | | 44% | 114 | Unknown conserved protein | 1.0e-14 | 68 | anopheles_darlingi_EST.fasta | FK703975 |
| GR486378 | 2 | | 50% | 153 | Unknown conserved protein | 2.0e-27 | 112 | anopheles_darlingi_EST.fasta | FK705369 |
| GR486329 | 1 | | 57% | 204 | Unknown conserved protein | 1.0e-26 | 113 | Anopheles_gambiae.AgamP3.50.pep.all.fa | AGAP007745-PA |
| GR486327 | 1 | | 50% | 189 | Unknown conserved protein | 1.0e-08 | 51 | anopheles_darlingi_EST.fasta | FK705188 |
| GR486400, GR486419 | 2 | | 49% | 957 | Unknown conserved protein with coiled-coil domain | 5.0e-12 | 78 | aaegypti.CONTIGS-Liverpool.AaegL1.fa | AAGE02021470.1 |
| GR486460 | 1 | | 56% | 159 | Unknown conserved protein | 5.0e-21 | 95 | Anopheles_gambiae.AgamP3.50.pep.all.fa | AGAP010723-PA |
| GR486512 | 1 | | 56% | 828 | Unknown conserved protein | 4.0e-11 | 47 | anopheles_darlingi_EST.fasta | FK704111 |
| GR486522 | 1 | | 51% | 441 | Unknown conserved protein | 7.0e-09 | 51 | agambiae.EST-CLIPPED.mar08.fa | BX615325.1 |
| GR486510 | 1 | | 58% | 147 | Unknown conserved protein | 2.0e-14 | 75 | Anopheles_gambiae.AgamP3.50.pep.all.fa | AGAP008438-PA |
| GR486441 | 1 | | 51% | 441 | Unknown conserved protein | 7.0e-09 | 51 | agambiae.EST-CLIPPED.mar08.fa | BX615325.1 |
| GR486485 | 2 | | 53% | 123 | Unknown conserved protein | 2.0e-16 | 79 | Anopheles_gambiae.AgamP3.50.pep.all.fa | AGAP012926-PA |
| GR486470 | 1 | | 51% | 477 | Unknown conserved protein | 4.0e-08 | 49 | agambiae.EST-CLIPPED.mar08.fa | BX615325.1 |
| GR486438 | 1 | | 44% | 204 | Unknown conserved protein | 5.0e-19 | 50 | anopheles_darlingi_EST.fasta | DV729753 |
| GR486440 | 1 | | 36% | 576 | Unknown conserved protein | 1.0e-44 | 84 | anopheles_darlingi_EST.fasta | DV729447 |
| GR486668 | 1 | | 52% | 468 | Unknown conserved protein | 4.0e-45 | 135 | anopheles_darlingi_EST.fasta | DV729843 |
| GR486634 | 1 | | 39% | 75 | Unknown conserved protein | 2.0e-18 | 62 | anopheles_darlingi_EST.fasta | FK704481 |
| GR486619 | 1 | | 37% | 84 | Unknown conserved protein | 5.0e-20 | 77 | anopheles_darlingi_EST.fasta | FK703921 |
| GR486574 | 3 | | 48% | 183 | Unknown conserved protein | 3.0e-28 | 118 | Anopheles_gambiae.AgamP3.50.pep.all.fa | AGAP002010-PA |
| GR486623 | 1 | | 52% | 402 | Unknown conserved protein with NUC173 domain | 2.0e-42 | 167 | Anopheles_gambiae.AgamP3.50.pep.all.fa | AGAP002961-PA |
| GR486536 | 2 | | 54% | 162 | Unknown conserved protein | 5.0e-13 | 36 | anopheles_darlingi_EST.fasta | FK705500 |
| GR486757 | 1 | | 70% | 141 | Unknown conserved protein | 5.0e-06 | 45 | cpipiens.EST-CLIPPED.mar08.fa | EV302468.1 |
| GR486691 | 1 | | 50% | 114 | Unknown conserved protein | 7.0e-10 | 60 | aaegypti.EST-CLIPPED.mar08.fa | DV322465.1 |
| **Bacterial protein** | | | | | | | | | |
| GR486501, GR487733 | 4 | | 57% | 87 | Bacterial protein | 1.0e-07 | 53 | refseq_protein | ZP_00630616 |
| GR486543 | 1 | | 53% | 78 | Bacterial protein | 4.0e-06 | 47 | refseq_protein | ZP_00630616 |
| GR486657 | 1 | | 72% | 225 | Bacterial protein | 1.0e-10 | 69 | uniprot_trembl.fasta | 25737_uniprot_trembl |
| GR486648 | 1 | | 55% | 78 | Bacterial protein | 2.0e-06 | 47 | refseq_protein | ZP_00630616 |
| GR486678 | 1 | | 49% | 168 | Bacterial protein | 3.0e-11 | 70 | uniref90.fasta | UniRef90_Q3BKI4 |
| GR486725 | 1 | | 53% | 194 | Bacterial protein | 8.0e-06 | 46 | uniref90.fasta | UniRef90_UPI00005545 |
| GR486773 | 1 | | 50% | 225 | Arabinose-proton symporter bacterial protein | 1.0e-14 | 82 | refseq_protein | ZP_02038208 |
| GR486780 | 1 | | 56% | 87 | Bacterial protein | 1.0e-07 | 53 | refseq_protein | ZP_00630616 |
