# Supplementary material for: Anopheles aquasalis Infected by Plasmodium vivax Displays Unique Gene Expression Profiles when Compared to Other Malaria Vectors and Plasmodia
Source: PLoS One. 2010 Mar 22;5(3):e9795. doi: 10.1371/journal.pone.0009795 (PMC2842430; doi:10.1371/journal.pone.0009795)
Supplement: Table S2 — List of sequences from the 2 hours infected minus non-infected insects library. Sequences with significant similarity on BLASTN or BLASTX were grouped based on the function of the homologous protein. (0.28 MB DOC) [file pone.0009795.s004.doc]

| **Accession number** | **Number of reads** | **G+C Content** | **CDS Length** | **Annotated Description** | **E-value** | **Score** | **Organism/Database** | **Gene**  **Accession**  **no.** |
| --- | --- | --- | --- | --- | --- | --- | --- | --- |
| **Signal transduction mechanism** | | | | | | | | |
| GR486807 | 2 | 52% | 327 | Phosrestin ii (arrestin N) | 6.0e-57 | 214 | Anopheles_gambiae.AgamP3.50.pep.all.fa | AGAP010134-PA |
| GR486806 | 1 | 64% | 108 | Rhodopsin receptor 3 | 3.0e-13 | 69 | Anopheles_gambiae.AgamP3.50.pep.all.fa | AGAP001178-PA |
| GR487139 | 1 | 54% | 183 | Adenylate and Guanylate cyclase | 3.0e-31 | 129 | Anopheles_gambiae.AgamP3.50.pep.all.fa | AGAP002998-PA |
| GR486987 | 1 | 56% | 189 | LIM domain-binding protein | 4.0e-37 | 148 | Anopheles_gambiae.AgamP3.50.pep.all.fa | AGAP006901-PC |
| **Biomolecules degradation** | | | | | | | | |
| GR486815 | 1 | 56% | 216 | Zinc carboxypeptidase A 1 | 3.0e-36 | 132 | Anopheles_gambiae.AgamP3.50.pep.all.fa | AGAP009593-PA |
| GR486794 | 1 | 62% | 168 | Zinc carboxypeptidase A 1 | 4.0e-16 | 79 | Anopheles_gambiae.AgamP3.50.pep.all.fa | AGAP009593-PA |
| GR486817 | 5 | 53% | 633 | Chymotrypsin-like protein | 1.0e-72 | 268 | Anopheles_gambiae.AgamP3.50.pep.all.fa | AGAP001198-PA |
| GR486928 | 1 | 53% | 165 | Fructose-1,6-bisphosphatase | 3.0e-25 | 108 | Anopheles_gambiae.AgamP3.50.pep.all.fa | AGAP009173-PA |
| GR486820 | 1 | 49% | 267 | Alpha-amylase | 2.0e-41 | 162 | Anopheles_gambiae.AgamP3.50.pep.all.fa | AGAP012401-PA |
| GR486808 | 1 | 49% | 153 | Chymotrypsin 1 | 9.0e-27 | 103 | anopheles_aquasalis_ptna.fasta | AAD17491 |
| GR486875 | 2 | 55% | 249 | Ubiquitin-activating enzyme E1 | 2.0e-40 | 159 | Anopheles_gambiae.AgamP3.50.pep.all.fa | AGAP011872-PA |
| GR486905 | 1 | 52% | 267 | Alpha-amylase / Maltase-like protein Agm2 | 3.0e-45 | 175 | Anopheles_gambiae.AgamP3.50.pep.all.fa | AGAP012400-PA |
| GR486957 | 2 | 57% | 114 | Chymotrypsin | 2.0e-10 | 60 | Anopheles_gambiae.AgamP3.50.pep.all.fa | AGAP010547-PA |
| GR486935 | 1 | 56% | 291 | Serine protease SP24D | 2.0e-26 | 112 | Anopheles_gambiae.AgamP3.50.pep.all.fa | AGAP005065-PA |
| GR487069 | 1 | 28% | 162 | Subtilisin-like serine protease | 2.0e-27 | 99 | anopheles_darlingi_EST.fasta | FK703921 |
| GR487148 | 1 | 56% | 249 | Ubiquitin-activating enzyme E1 | 3.0e-41 | 161 | Anopheles_gambiae.AgamP3.50.pep.all.fa | AGAP011872-PA |
| GR487150 | 1 | 53% | 219 | Protease M1 zinc metalloprotease | 4.0e-08 | 52 | Anopheles_gambiae.AgamP3.50.pep.all.fa | AGAP012745-PA |
| GR487054 | 1 | 57% | 228 | Insulinase family metalloproteinase | 6.0e-32 | 130 | Anopheles_gambiae.AgamP3.50.pep.all.fa | AGAP006099-PA |
| GR487131 | 2 | 49% | 204 | Metalloprotease | 8.0e-22 | 97 | Anopheles_gambiae.AgamP3.50.pep.all.fa | AGAP004747-PA |
| GR486970 | 1 | 60% | 96 | Ubiquinol-cytochrome c reductase complex core protein | 4.0e-10 | 59 | Anopheles_gambiae.AgamP3.50.pep.all.fa | AGAP006099-PA |
| GR487046 | 1 | 61% | 96 | Insulinase (Peptidase family M16) | 2.0e-10 | 59 | Anopheles_gambiae.AgamP3.50.pep.all.fa | AGAP006099-PA |
| GR487040 | 3 | 54% | 330 | Chymotrypsin A | 1.0e-34 | 139 | Anopheles_gambiae.AgamP3.50.pep.all.fa | AGAP001199-PA |
| GR486963 | 1 | 57% | 183 | Hsp70 chaperones | 2.0e-28 | 119 | cpipiens.PEPTIDES-CpipJ1.1.fa | CPIJ008915-PA |
| GR486979 | 1 | 58% | 114 | Chymotrypsin | 3.0e-10 | 59 | Anopheles_gambiae.AgamP3.50.pep.all.fa | AGAP010547-PA |
| **Replication, translation and transcription** | | | | | | | | |
| GR486798 | 2 | 59% | 405 | 60S ribosomal protein L21 | 1.0e-75 | 270 | anopheles_darlingi_ptna.fasta | ACI30091 |
| GR486793 | 1 | 50% | 345 | Proliferating Cell Nuclear Antigen (PCNA) | 2.0e-57 | 215 | Anopheles_gambiae.AgamP3.50.pep.all.fa | AGAP010220-PA |
| GR486810 | 1 | 40% | 564 | Extensin 2 | 2.0e-16 | 86 | aaegypti.EST-CLIPPED.mar08.fa EG009251.1 | EG009251 |
| GR486879 | 1 | 55% | 255 | 5' nucleotidase apyrase | 2.0e-43 | 162 | anopheles_darlingi_ptna.fasta | ACI30113 |
| GR486831 | 1 | 59% | 183 | 40S ribosomal protein S23 | 5.0e-30 | 117 | anopheles_darlingi_ptna.fasta | ACI30052 |
| GR486969 | 2 | 57% | 93 | 60S ribosomal protein L10 | 1.0e-13 | 70 | Anopheles_gambiae.AgamP3.50.pep.all.fa | AGAP011298-PA |
| GR486986 | 38 | 28% | 276 | Mitochondrial large ribosomal RNA | 4.0e-36 | 149 | dmel-all-gene-r5.8.fasta | FBgn0013686 |
| GR486974 | 1 | 57% | 75 | 60S ribosomal protein L22 | 1.0e-10 | 53 | anopheles_darlingi_ptna.fasta | ACI30068 |
| GR486882 | 1 | 66% | 198 | 60S ribosomal protein L7a | 3.0e-20 | 86 | anopheles_darlingi_ptna.fasta | ACI30081 |
| GR486956 | 1 | 61% | 342 | 40S ribosomal protein S2/30S ribosomal protein S5 | 1.0e-58 | 212 | Anopheles_gambiae.AgamP3.50.pep.all.fa | AGAP003768-PA |
| GR487127 | 1 | 51% | 174 | Ribosomal protein L22 | 3.0e-07 | 43 | anopheles_darlingi_nucleotideo.fasta | EU934314 |
| GR487124 | 1 | 55% | 294 | 60S ribosomal protein L24 | 1.0e-21 | 91 | anopheles_darlingi_ptna.fasta | ACI30074 |
| GR487092 | 2 | 53% | 240 | DNA/RNA repair protein | 5.0e-38 | 151 | Anopheles_gambiae.AgamP3.50.pep.all.fa | AGAP002472-PA |
| GR487026 | 7 | 55% | 213 | 60S ribosomal protein L5 | 6.0e-30 | 124 | cpipiens.PEPTIDES-CpipJ1.1.fa | CPIJ010112-PA |
| GR487048 | 2 | 54% | 216 | RUVB-related reptin and pontin | 2.0e-33 | 132 | Anopheles_gambiae.AgamP3.50.pep.all.fa | AGAP009746-PA |
| GR486866 | 1 | 55% | 102 | Ubiquitin/60S ribosomal protein L40 fusion | 4.0e-15 | 68 | anopheles_darlingi_ptna.fasta | ACI30049 |
| GR486949 | 1 | 52% | 195 | Homeobox protein extradenticle | 3.0e-27 | 115 | Anopheles_gambiae.AgamP3.50.pep.all.fa | AGAP004696-PA |
| GR486983 | 1 | 62% | 147 | Translation elongation factor 2 | 2.0e-28 | 89 | Anopheles_gambiae.AgamP3.50.pep.all.fa | AGAP009441-PA |
| GR487063 | 1 | 58% | 186 | Elongation factor 1-gamma | 6.0e-32 | 130 | Anopheles_gambiae.AgamP3.50.pep.all.fa | AGAP000883-PA |
| GR486868 | 2 | 59% | 231 | Eukaryotic translation elongation factor | 7.0e-59 | 154 | Anopheles_gambiae.AgamP3.50.pep.all.fa | AGAP009441-PA |
| GR487025 | 4 | 47% | 504 | Homeobox protein extradenticle | 6.0e-89 | 322 | Anopheles_gambiae.AgamP3.50.pep.all.fa | AGAP004696-PA |
| GR487081 | 2 | 54% | 165 | Mediator of RNA polymerase II transcription subunit 22 | 2.0e-22 | 100 | Anopheles_gambiae.AgamP3.50.pep.all.fa | AGAP004191-PA |
| **Metabolism** | | | | | | | | |
| GR487021 | 1 | 61% | 138 | Creatine kinase | 6.0e-20 | 91 | Anopheles_gambiae.AgamP3.50.pep.all.fa | AGAP005627-PB |
| GR487113 | 1 | 46% | 279 | Coproporphyrinogen III oxidase | 6.0e-49 | 187 | Anopheles_gambiae.AgamP3.50.pep.all.fa | AGAP004749-PB |
| GR486965 | 1 | 62% | 249 | Bifunctional purine biosynthesis protein | 1.0e-37 | 150 | Aedes_aegypti.AaegL1.50.pep.all.fa | AAEL012825-PA |
| GR487008 | 3 | 58% | 318 | Fatty acid desaturase | 2.0e-59 | 222 | Anopheles_gambiae.AgamP3.50.pep.all.fa | AGAP001713-PA |
| GR487129 | 2 | 52% | 270 | Ribose-phosphate pyrophosphokinase | 5.0e-48 | 185 | Anopheles_gambiae.AgamP3.50.pep.all.fa | AGAP004890-PB |
| GR486994 | 1 | 55% | 66 | Haloacid dehalogenase-like hydrolase | 6.0e-06 | 45 | Anopheles_gambiae.AgamP3.50.pep.all.fa | AGAP002841-PA |
| **Defense and detoxification** | | | | | | | | |
| GR486800 | 2 | 61% | 150 | Bacteria responsive protein 2 / imaginal disc growth factor | 5.0e-22 | 98 | Anopheles_gambiae.AgamP3.50.pep.all.fa | AGAP008060-PA |
| GR486898 | 1 | 55% | 138 | Fibronectin | 5.0e-21 | 95 | Anopheles_gambiae.AgamP3.50.pep.all.fa | AGAP002579-PA |
| GR487115 | 1 | 54% | 96 | Alpha-2-macroglobulin receptor-associated protein | 1.0e-14 | 56 | Anopheles_gambiae.AgamP3.50.pep.all.fa | AGAP003521-PA |
| GR487133 | 1 | 42% | 336 | Fibrinogen (ficolin) | 1.0e-19 | 90 | Anopheles_gambiae.AgamP3.50.pep.all.fa | AGAP010811-PA |
| GR487128 | 3 | 51% | 141 | Alpha-2-macroglobulin receptor-associated protein | 3.0e-21 | 96 | Anopheles_gambiae.AgamP3.50.pep.all.fa | AGAP003521-PA |
| **Structural genes** | | | | | | | | |
| GR486812 | 3 | 47% | 99 | Profilin | 3.0e-13 | 69 | Anopheles_gambiae.AgamP3.50.pep.all.fa | AGAP009861-PA |
| GR486936 | 3 | 58% | 276 | Actin | 2.0e-47 | 182 | Aedes_aegypti.AaegL1.50.pep.all.fa | AAEL004631-PA |
| GR486917 | 3 | 57% | 189 | Actin | 3.0e-34 | 138 | Anopheles_gambiae.AgamP3.50.pep.all.fa | AGAP005095-PA |
| GR486912 | 3 | 53% | 129 | Myotonin-protein kinase | 1.0e-18 | 87 | Anopheles_gambiae.AgamP3.50.pep.all.fa | AGAP012090-PA |
| GR486947 | 2 | 49% | 147 | Cuticular protein 76 | 4.0e-19 | 89 | Anopheles_gambiae.AgamP3.50.pep.all.fa | AGAP009874-PA |
| GR486927 | 1 | 47% | 237 | Actin |  |  |  |  |
| GR487080 | 1 | 56% | 147 | Collagen IV alpha 1 chain | 5.0e-25 | 108 | cpipiens.PEPTIDES-CpipJ1.1.fa | CPIJ005294-PA |
| GR487057 | 1 | 57% | 138 | Actin | 1.0e-21 | 97 | Aedes_aegypti.AaegL1.50.pep.all.fa | AAEL005961-PA |
| GR487024 | 1 | 55% | 339 | Laminin subunit gamma-1 | 5.0e-27 | 114 | Anopheles_gambiae.AgamP3.50.pep.all.fa | AGAP007629-PA |
| **Energy metabolism** | | | | | | | | |
| GR486805 | 2 | 56% | 195 | Fumarylacetoacetate hydrolase | 6.0e-31 | 127 | Anopheles_gambiae.AgamP3.50.pep.all.fa | AGAP005865-PA |
| GR486939 | 2 | 56% | 258 | Succinyl-coa:3-ketoacid-coenzyme a transferase | 2.0e-43 | 169 | Anopheles_gambiae.AgamP3.50.pep.all.fa | AGAP006096-PA |
| GR486946 | 2 | 56% | 174 | Mitochondrial phosphate carrier protein | 1.0e-29 | 123 | Anopheles_gambiae.AgamP3.50.pep.all.fa | AGAP003586-PA |
| GR486887 | 2 | 45% | 210 | Succinyl-coa synthetase beta chain | 1.0e-33 | 136 | Aedes_aegypti.AaegL1.50.pep.all.fa | AAEL011746-PA |
| GR487019 | 2 | 61% | 75 | 3-hydroxyacyl-coa dehydrogenase | 1.0e-06 | 48 | Anopheles_gambiae.AgamP3.50.pep.all.fa | AGAP007784-PA |
| GR487121 | 1 | 63% | 75 | Multifunctional fatty acid oxidation complex | 9.0e-06 | 45 | Anopheles_gambiae.AgamP3.50.pep.all.fa | AGAP007784-PA |
| GR487114 | 1 | 51% | 246 | Glucosidase II beta subunit-like protein | 6.0e-31 | 127 | Anopheles_gambiae.AgamP3.50.pep.all.fa | AGAP009546-PA |
| GR487090 | 2 | 54% | 273 | S-adenosylmethionine synthetase | 1.0e-21 | 97 | Anopheles_gambiae.AgamP3.50.pep.all.fa | AGAP009447-PA |
| GR487137 | 1 | 55% | 165 | Mitochondrial Aconitase | 1.0e-26 | 113 | Anopheles_gambiae.AgamP3.50.pep.all.fa | AGAP007852-PA |
| GR487100 | 1 | 51% | 210 | Succinyl-coa synthetase beta chain | 5.0e-18 | 85 | Anopheles_gambiae.AgamP3.50.pep.all.fa | AGAP004744-PA |
| GR487117 | 1 | 62% | 126 | Cytochrome c oxidase subunit IV | 1.0e-17 | 84 | Anopheles_gambiae.AgamP3.50.pep.all.fa | AGAP008727-PA |
| GR487116 | 1 | 52% | 312 | Phosphorylase kinase alpha/beta | 1.0e-39 | 156 | Anopheles_gambiae.AgamP3.50.pep.all.fa | AGAP009278-PA |
| GR486860 | 3 | 52% | 243 | Dihydrolipoamide succinyltransferase | 7.0e-39 | 154 | Aedes_aegypti.AaegL1.50.pep.all.fa | AAEL002764-PB |
| **Embryogenesis** | | | | | | | | |
| GR486841 | 10 | 54% | 231 | Vitellogenin | 3.0e-16 | 87 | uniref90.fasta | UniRef90_Q49MF2 |
| GR486827 | 1 | 61% | 186 | Apolipophorins / vitellogenin | 3.0e-19 | 89 | Anopheles_gambiae.AgamP3.50.pep.all.fa | AGAP001826-PA |
| GR487097 | 1 | 55% | 237 | Vitellogenin | 1.0e-39 | 164 | uniref90.fasta | UniRef90_Q49MF2 |
| GR487034 | 2 | 59% | 285 | Apolipophorins | 3.0e-43 | 168 | Anopheles_gambiae.AgamP3.50.pep.all.fa | AGAP001826-PA |
| GR486937 | 1 | 52% | 147 | Maternal protein exuperantia | 2.0e-21 | 96 | Aedes_aegypti.AaegL1.50.pep.all.fa | AAEL010097-PA |
| **Transport and secretion** | | | | | | | | |
| GR486819 | 2 | 54% | 123 | Ferritin light chain-like protein precursor | 1.0e-14 | 67 | anopheles_darlingi_ptna.fasta | ACI30191 |
| GR486929 | 1 | 52% | 315 | Ferritin heavy chain | 2.0e-27 | 115 | Anopheles_gambiae.AgamP3.50.pep.all.fa | AGAP002465-PA |
| GR486950 | 1 | 43% | 75 | Amino acid permease | 4.0e-07 | 49 | Anopheles_gambiae.AgamP3.50.pep.all.fa | AGAP011386-PA |
| GR487007 | 1 | 55% | 303 | Importin alpha | 5.0e-45 | 174 | Anopheles_gambiae.AgamP3.50.pep.all.fa | AGAP001273-PA |
| GR487035 | 3 | 52% | 522 | Importin alpha | 5.0e-90 | 325 | Anopheles_gambiae.AgamP3.50.pep.all.fa | AGAP001273-PA |
| GR486988 | 1 | 58% | 117 | Pmp22 peroxisomal membrane protein | 1.0e-14 | 74 | Aedes_aegypti.AaegL1.50.pep.all.fa | AAEL004577-PA |
| GR486894 | 1 | 56% | 135 | Nuclear pore complex protein Nup107 | 1.0e-18 | 87 | Anopheles_gambiae.AgamP3.50.pep.all.fa | AGAP001685-PA |
| GR486954 | 1 | 54% | 198 | Importin alpha | 2.0e-25 | 109 | Anopheles_gambiae.AgamP3.50.pep.all.fa | AGAP001273-PA |
| GR487027 | 5 | 59% | 309 | Calcium-transporting ATPase sarcoplasmic/endoplasmic reticulum type | 8.0e-54 | 203 | Anopheles_gambiae.AgamP3.50.pep.all.fa | AGAP006186-PD |
| GR487052 | 4 | 49% | 198 | Retinal degeneration b beta | 5.0e-33 | 135 | cpipiens.PEPTIDES-CpipJ1.1.fa | CPIJ009191-PA |
| **Unknown protein** | | | | | | | | |
| GR486816 | 2 | 44% | 375 | Unknown protein |  |  |  |  |
| GR486802 | 1 | 49% | 315 | Unknown protein |  |  |  |  |
| GR486797 | 1 | 53% | 114 | Unknown protein |  |  |  |  |
| GR486930 | 1 | 43% | 330 | Unknown protein |  |  |  |  |
| GR486931 | 1 | 48% | 162 | Unknown protein |  |  |  |  |
| GR486845 | 1 | 40% | 339 | Unknown protein |  |  |  |  |
| GR486811 | 1 | 42% | 468 | Unknown protein |  |  |  |  |
| GR486821 | 1 | 40% | 240 | Unknown protein |  |  |  |  |
| GR486828 | 1 | 48% | 228 | Unknown protein |  |  |  |  |
| GR487154 | 1 | 50% | 192 | Unknown protein |  |  |  |  |
| GR487161 | 1 | 46% | 291 | Unknown protein |  |  |  |  |
| GR486899 | 61 | 52% | 498 | Unknown protein |  |  |  |  |
| GR486854 | 1 | 50% | 384 | Unknown protein |  |  |  |  |
| GR486952 | 1 | 49% | 183 | Unknown protein |  |  |  |  |
| GR487056 | 1 | 32% | 183 | Unknown protein |  |  |  |  |
| GR486863 | 1 | 53% | 180 | Unknown protein |  |  |  |  |
| GR486964 | 1 | 56% | 240 | Unknown protein |  |  |  |  |
| GR486903 | 1 | 56% | 141 | Unknown protein |  |  |  |  |
| GR486862 | 1 | 43% | 522 | Unknown protein |  |  |  |  |
| GR486865 | 2 | 51% | 438 | Unknown protein |  |  |  |  |
| GR486942 | 1 | 51% | 243 | Unknown protein |  |  |  |  |
| GR486980 | 2 | 49% | 168 | Unknown protein |  |  |  |  |
| GR486891 | 5 | 48% | 150 | Unknown protein |  |  |  |  |
| GR486995 | 1 | 51% | 345 | Unknown protein |  |  |  |  |
| GR486967 | 1 | 17% | 180 | Unknown protein with coiled-coil domain |  |  |  |  |
| GR486848 | 1 | 42% | 459 | Unknown protein |  |  |  |  |
| GR487079 | 1 | 44% | 207 | Unknown protein |  |  |  |  |
| GR487041 | 1 | 44% | 378 | Unknown protein |  |  |  |  |
| GR486921 | 2 | 52% | 246 | Unknown protein |  |  |  |  |
| GR486872, GR486977 | 2 | 44% | 522 | Unknown protein |  |  |  |  |
| GR486960 | 1 | 44% | 213 | Unknown protein |  |  |  |  |
| GR487047 | 1 | 49% | 183 | Unknown protein |  |  |  |  |
| GR486889 | 3 | 48% | 288 | Unknown protein |  |  |  |  |
| GR486858 | 1 | 32% | 183 | Unknown protein |  |  |  |  |
| GR487125 | 1 | 41% | 192 | Unknown protein |  |  |  |  |
| GR487029 | 6 | 51% | 174 | Unknown protein / ribosomal protein L22 |  |  |  |  |
| GR487006 | 2 | 47% | 135 | Unknown protein |  |  |  |  |
| GR487077 | 1 | 49% | 162 | Unknown protein |  |  |  |  |
| GR487152 | 6 | 42% | 441 | Unknown protein |  |  |  |  |
| GR487106 | 1 | 44% | 210 | Unknown protein |  |  |  |  |
| GR487083 | 1 | 49% | 339 | Unknown protein |  |  |  |  |
| GR487134 | 1 | 45% | 198 | Unknown protein |  |  |  |  |
| GR486989 | 1 | 50% | 129 | Unknown protein |  |  |  |  |
| GR487039 | 1 | 45% | 243 | Unknown protein |  |  |  |  |
| GR487017 | 1 | 33% | 174 | Unknown protein |  |  |  |  |
| GR487144 | 1 | 50% | 327 | Unknown protein |  |  |  |  |
| GR487020 | 1 | 52% | 168 | Unknown protein |  |  |  |  |
| GR487142 | 1 | 34% | 180 | Unknown protein |  |  |  |  |
| GR487013 | 1 | 44% | 321 | Unknown protein |  |  |  |  |
| GR487143 | 1 | 48% | 420 | Unknown protein |  |  |  |  |
| GR487051 | 1 | 52% | 132 | Unknown protein |  |  |  |  |
| GR487078 | 1 | 48% | 96 | Unknown protein |  |  |  |  |
| GR487109 | 3 | 45% | 432 | Unknown protein |  |  |  |  |
| GR487055 | 1 | 43% | 228 | Unknown protein |  |  |  |  |
| GR487105 | 1 | 48% | 420 | Unknown protein |  |  |  |  |
| GR487003 | 1 | 57% | 138 | Unknown protein |  |  |  |  |
| GR487107 | 1 | 47% | 336 | Unknown protein |  |  |  |  |
| GR486998 | 2 | 50% | 339 | Unknown protein |  |  |  |  |
| GR486958 | 1 | 50% | 96 | Unknown protein |  |  |  |  |
| GR486968 | 1 | 50% | 222 | Unknown protein |  |  |  |  |
| GR487049 | 1 | 51% | 153 | Unknown protein |  |  |  |  |
| GR487060 | 1 | 44% | 174 | Unknown protein |  |  |  |  |
| GR487111 | 1 | 48% | 180 | Unknown protein |  |  |  |  |
| GR486886 | 2 | 49% | 147 | Unknown protein |  |  |  |  |
| GR487110 | 1 | 32% | 180 | Unknown protein |  |  |  |  |
| **Unknown conserved protein** | | | | | | | | |
| GR486838 | 1 | 59% | 141 | Unknown conserved protein | 1.0e-11 | 74 | aaegypti.CONTIGS-Liverpool.AaegL1.fa | AAGE02020711.1 |
| GR486823 | 1 | 52% | 63 | Unknown conserved protein | 4.0e-06 | 49 | aegypti.EST-CLIPPED.mar08.fa | DV370849.1 |
| GR486871 | 1 | 51% | 135 | Unknown conserved protein | 2.0e-09 | 52 | anopheles_darlingi_EST.fasta | DV729374 |
| GR486918 | 1 | 49% | 87 | Unknown conserved protein | 1.0e-10 | 55 | aaegypti.EST-CLIPPED.mar08.fa | DV321842.1 |
| GR487160 | 1 | 48% | 81 | Unknown conserved protein | 3.0e-10 | 54 | aaegypti.EST-CLIPPED.mar08.fa | DV322465.1 |
| GR486878 | 1 | 50% | 111 | Unknown conserved protein | 2.0e-12 | 68 | aaegypti.EST-CLIPPED.mar08.fa | DV322465.1 |
| GR487009 | 1 | 53% | 153 | Unknown conserved protein | 2.0e-08 | 56 | raaegypti.EST-CLIPPED.mar08.fa | DV321842.1 |
| GR487172 | 1 | 58% | 156 | Unknown conserved protein | 2.0e-11 | 58 | aaegypti.EST-CLIPPED.mar08.fa | DV321842.1 |
| GR486940 | 1 | 37% | 261 | Unknown conserved protein with coiled-coil domain | 2.0e-15 | 50 | anopheles_darlingi_EST.fasta | FK704481 |
| GR486984 | 2 | 45% | 156 | Unknown conserved protein with Leucine-rich repeat (LRR) | 2.0e-06 | 47 | Anopheles_gambiae.AgamP3.50.pep.all.fa | AGAP007453-PA |
| GR487075 | 1 | 52% | 417 | Unknown conserved protein | 4.0e-64 | 238 | Anopheles_gambiae.AgamP3.50.pep.all.fa | AGAP007851-PA |
| GR487044 | 1 | 48% | 162 | Unknown conserved protein | 4.0e-08 | 52 | Anopheles_gambiae.AgamP3.50.pep.all.fa | AGAP003939-PA |
| GR486907 | 2 | 42% | 389 | Unknown conserved protein | 2.0e-35 | 83 | anopheles_darlingi_EST.fasta | FK704163 |
| GR486938 | 2 | 43% | 387 | Unknown conserved protein | 6.0e-28 | 118 | Anopheles_gambiae.AgamP3.50.pep.all.fa | AGAP006275-PA |
| GR487122 | 1 | 45% | 111 | Unknown conserved protein | 4.0e-07 | 53 | cpipiens.SUPERCONTIGS-Johannesburg.CpipJ1.fa | DS231941.1 |
| GR486962 | 1 | 50% | 231 | Unknown conserved protein | 2.0e-08 | 57 | agambiae.EST-CLIPPED.mar08.fa | BX605447.1 |
| GR487099 | 1 | 50% | 294 | Unknown conserved protein | 2.0e-11 | 62 | rprolixus.EST-CLIPPED.mar08.fa FD777574.1 | FD777574 |
| GR486892 | 1 | 55% | 351 | Unknown conserved protein | 2.0e-47 | 183 | Anopheles_gambiae.AgamP3.50.pep.all.fa | AGAP011476-PA |
| **Bacterial protein** | | | | | | | | |
| GR487168 | 1 | 55% | 192 | Bacterial protein | 2.0e-09 | 59 | uniref90.fasta | UniRef90_UPI00005545 |
| GR487162 | 1 | 56% | 87 | Bacterial protein |  |  |  |  |
