# Supplementary material for: Anopheles aquasalis Infected by Plasmodium vivax Displays Unique Gene Expression Profiles when Compared to Other Malaria Vectors and Plasmodia
Source: PLoS One. 2010 Mar 22;5(3):e9795. doi: 10.1371/journal.pone.0009795 (PMC2842430; doi:10.1371/journal.pone.0009795)
Supplement: Table S3 — List of sequences from the 24 hours non-infected minus infected insects library. Sequences with significant similarity on BLASTN or BLASTX were grouped based on the function of the homologous protein. (0.13 MB DOC) [file pone.0009795.s005.doc]

| **Accession number** | **Number of reads** | | **G+C Content** | | **CDS Length** | | **Annotated Description** | | **E-value** | | **Score** | | **Organism/Database** | | **Gene**  **Accession**  **no.** | |
| --- | --- | --- | --- | --- | --- | --- | --- | --- | --- | --- | --- | --- | --- | --- | --- | --- |
| **Signal transduction mechanism** | | | | | | | | | | | | | | | | |
| GR487233 | 1 | | 53% | | 168 | | Rhodopsin receptor 1 | | 6.0e-19 | | 88 | | Anopheles_gambiae.AgamP3.50.pep.all.fa | | AGAP001178-PA | |
| GR487190 | 1 | | 57% | | 114 | | Ultraviolet-sensitive opsin | | 3.0e-29 | | 57 | | cpipiens.PEPTIDES-CpipJ1.1.fa | | CPIJ013408-PA | |
| GR487344 | 1 | | 60% | | 171 | | Rhodopsin receptor 1 | | 3.0e-25 | | 108 | | Anopheles_gambiae.AgamP3.50.pep.all.fa | | AGAP001178-PA | |
| GR487351 | 3 | | 64% | | 108 | | Rhodopsin receptor 3 | | 3.0e-13 | | 69 | | Anopheles_gambiae.AgamP3.50.pep.all.fa | | AGAP001178-PA | |
| **Biomolecules degradation** | | | | | | | | | | | | | | | | |
| GR487200 | 5 | | 50% | | 165 | | Chymotrypsin 1 | | 1.0e-28 | | 110 | | anopheles_aquasalis_ptna.fasta | | AAD17491 | |
| GR487219 | 1 | | 61% | | 135 | | Cathepsin 1 | | 3.0e-21 | | 96 | | Anopheles_gambiae.AgamP3.50.pep.all.fa | | AGAP012577-PA | |
| GR487348 | 3 | | 62% | | 90 | | Cathepsin 1 | | 7.0e-13 | | 68 | | Anopheles_gambiae.AgamP3.50.pep.all.fa | | AGAP011828-PA | |
| GR487377 | 1 | | 56% | | 126 | | Chymotrypsin 1 | | 2.0e-23 | | 93 | | anopheles_aquasalis_ptna.fasta | | AAD17491 | |
| GR487341 | 15 | | 57% | | 126 | | Chymotrypsin 1 | | 2.0e-23 | | 93 | | anopheles_aquasalis_ptna.fasta | | AAD17491 | |
| GR487388 | 1 | | 43% | | 192 | | Rhomboid 1 (intramembrane serine protease) | | 2.0e-21 | | 96 | | Anopheles_gambiae.AgamP3.50.pep.all.fa | | AGAP000832-PA | |
| **Replication, translation and transcription** | | | | | | | | | | | | | | | | |
| GR487203 | 1 | | 57% | | 69 | | Translationally-controlled tumor protein | | 4.0e-08 | | 52 | | Anopheles_gambiae.AgamP3.50.pep.all.fa | | AGAP002667-PA | |
| GR487236 | 4 | | 55% | | 294 | | 60S ribosomal protein L24 | | 1.0e-21 | | 91 | | anopheles_darlingi_ptna.fasta | | ACI30074 | |
| GR487363 | 1 | | 54% | | 96 | | 60S ribosomal protein L10 | | 4.0e-11 | | 62 | | Anopheles_gambiae.AgamP3.50.pep.all.fa | | AGAP011298-PA | |
| GR487217 | 2 | | 56% | | 165 | | 40S ribosomal protein S5 | | 3.0e-13 | | 69 | | Anopheles_gambiae.AgamP3.50.pep.all.fa | | AGAP008329-PA | |
| GR487235 | 12 | | 57% | | 93 | | 60S ribosomal protein L10 | | 1.0e-13 | | 70 | | Anopheles_gambiae.AgamP3.50.pep.all.fa | | AGAP011298-PA | |
| GR487342 | 4 | | 50% | | 261 | | DEAD box ATP-dependent RNA helicase | | 2.0e-35 | | 142 | | Anopheles_gambiae.AgamP3.50.pep.all.fa | | AGAP009863-PA | |
| GR487335 | 2 | | 58% | | 198 | | 40S ribosomal protein S4 | | 8.0e-35 | | 133 | | anopheles_darlingi_ptna.fasta | | ACI30066 | |
| **Metabolism** | | | | | | | | | | | | | | | | |
| GR487184 | 1 | | 61% | | 138 | | Creatine kinase | | 6.0e-20 | | 91 | | Anopheles_gambiae.AgamP3.50.pep.all.fa | | AGAP005627-PB | |
| GR487358 | 3 | | 57% | | 162 | | Phosphoserine phosphatase | | 4.0e-22 | | 99 | | Anopheles_gambiae.AgamP3.50.pep.all.fa | | AGAP012247-PA | |
| GR487209 | 1 | | 54% | | 321 | | Dimethylaniline monooxygenase | | 2.0e-54 | | 205 | | Anopheles_gambiae.AgamP3.50.pep.all.fa | | AGAP010398-PA | |
| **Defense and detoxification** | | | | | | | | | | | | | | | | |
| GR487220 | 1 | | 57% | | 114 | | Cu-Zn Superoxide Dismutase | | 1.0e-07 | | 52 | | Anopheles_gambiae.AgamP3.50.pep.all.fa | | AGAP001623-PA | |
| **Structural genes** | | | | | | | | | | | | | | | | |
| GR487244 | 2 | | 57% | | 297 | | Fibulin 1 | | 1.0e-48 | | 186 | | Anopheles_gambiae.AgamP3.50.pep.all.fa | | AGAP011322-PA | |
| **Energy metabolism** | | | | | | | | | | | | | | | | |
| GR487211 | 2 | | 55% | | 168 | | Cytochrome c oxidase subunit IV | | 3.0e-17 | | 83 | | Aedes_aegypti.AaegL1.50.pep.all.fa | | AAEL005170-PA | |
| **Embryogenesis** | | | | | | | | | | | | | | | | |
| GR487194 | 4 | | 56% | | 231 | | Vitellogenin | | 7.0e-14 | | 79 | | uniref90.fasta | | UniRef90_Q49MF2 | |
| GR487227 | 1 | | 56% | | 153 | | Vitellogenin | | 1.0e-25 | | 110 | | Anopheles_gambiae.AgamP3.50.pep.all.fa | | AGAP004203-PB | |
| GR487240 | 1 | | 59% | | 99 | | Vitellogenin | | 1.0e-12 | | 67 | | Anopheles_gambiae.AgamP3.50.pep.all.fa | | AGAP004203-PB | |
| GR487183 | 1 | | 57% | | 234 | | Vitellogenin | | 4.0e-08 | | 60 | | uniref90.fasta | | UniRef90_Q49MF2 | |
| GR487365 | 1 | | 56% | | 232 | | Vitellogenin | | 7.0e-14 | | 79 | | uniref90.fasta | | UniRef90_Q49MF2 | |
| GR487376 | 1 | | 57% | | 231 | | Vitellogenin | | 6.0e-13 | | 76 | | uniref90.fasta | | UniRef90_Q49MF2 | |
| GR487332 | 35 | | 54% | | 231 | | Vitellogenin | | 3.0e-16 | | 87 | | uniref90.fasta | | UniRef90_Q49MF2 | |
| GR487338 | 1 | | 57% | | 246 | | Vitellogenin | | 3.0e-15 | | 84 | | uniref90.fasta | | UniRef90_Q49MF2 | |
| GR487251 | 1 | | 62% | | 180 | | Vitellogenin | | 1.0e-06 | | 47 | | uniref90.fasta | | UniRef90_Q49MF2 | |
| GR487242 | 1 | | 56% | | 237 | | Vitellogenin | | 7.0e-15 | | 82 | | uniref90.fasta | | UniRef90_Q49MF2 | |
| GR487247 | 1 | | 57% | | 246 | | Vitellogenin | | 3.0e-15 | | 84 | | uniref90.fasta | | UniRef90_Q49MF2 | |
| GR487375 | 1 | | 62% | | 141 | | Vitellogenin | | 2.0e-09 | | 57 | | Anopheles_gambiae.AgamP3.50.pep.all.fa | | AGAP004203-PB | |
| GR487340 | 14 | | 59% | | 132 | | Vitellogenin | | 7.0e-20 | | 91 | | Anopheles_gambiae.AgamP3.50.pep.all.fa | | AGAP004203-PB | |
| GR487396 | 1 | | 56% | | 231 | | Vitellogenin | | 1.0e-12 | | 75 | | uniref90.fasta | | UniRef90_Q49MF2 | |
| GR487414 | 1 | | 57% | | 246 | | Vitellogenin | | 3.0e-13 | | 77 | | uniref90.fasta | | UniRef90_Q49MF2 | |
| GR487438 | 1 | | 60% | | 141 | | Vitellogenin | | 2.0e-19 | | 90 | | Anopheles_gambiae.AgamP3.50.pep.all.fa | | AGAP001826-PA | |
| GR487428 | 1 | | 55% | | 231 | | Vitellogenin | | 2.0e-13 | | 78 | | uniref90.fasta | | UniRef90_Q49MF2 | |
| GR487410 | 1 | | 57% | | 246 | | Vitellogenin | | 1.0e-14 | | 81 | | uniref90.fasta | | UniRef90_Q49MF2 | |
| GR487431 | 1 | | 57% | | 246 | | Vitellogenin | | 4.0e-14 | | 80 | | uniref90.fasta | | UniRef90_Q49MF2 | |
| GR487394 | 1 | | 55% | | 219 | | Vitellogenin | | 3.0e-12 | | 74 | | uniref90.fasta | | UniRef90_Q49MF2 | |
| GR487440 | 1 | | 55% | | 219 | | Vitellogenin | | 6.0e-11 | | 69 | | uniref90.fasta | | UniRef90_Q49MF2 | |
| GR487444 | 1 | | 58% | | 231 | | Vitellogenin | | 4.0e-11 | | 70 | | uniref90.fasta | | UniRef90_Q49MF2 | |
| **Unknown protein** | | | | | | | | | | | | | | | | |
| GR487197 | 1 | | 44% | | 207 | | Unknown protein | |  | |  | |  | |  | |
| GR487192 | 1 | | 52% | | 159 | | Unknown protein | |  | |  | |  | |  | |
| GR487255 | 4 | | 46% | | 420 | | Unknown protein | |  | |  | |  | |  | |
| GR487204 | 1 | | 53% | | 177 | | Unknown protein | |  | |  | |  | |  | |
| GR487287 | 1 | | 56% | | 189 | | Unknown protein | |  | |  | |  | |  | |
| GR487271 | 2 | | 53% | | 204 | | Unknown protein | |  | |  | |  | |  | |
| GR487199 | 3 | | 51% | | 174 | | Unknown protein / ribosomal protein L22 | |  | |  | |  | |  | |
| GR487260 | 1 | | 45% | | 183 | | Unknown protein | |  | |  | |  | |  | |
| GR487262 | 5 | | 48% | | 198 | | Unknown protein | |  | |  | |  | |  | |
| GR487368 | 1 | | 48% | | 201 | | Unknown protein | |  | |  | |  | |  | |
| GR487253 | 1 | | 50% | | 258 | | Unknown protein | |  | |  | |  | |  | |
| GR487221 | 1 | | 61% | | 207 | | Unknown protein | |  | |  | |  | |  | |
| GR487216 | 1 | | 46% | | 108 | | Unknown protein | |  | |  | |  | |  | |
| GR487369 | 1 | | 50% | | 363 | | Unknown protein | |  | |  | |  | |  | |
| GR487207 | 1 | | 49% | | 336 | | Unknown protein | |  | |  | |  | |  | |
| GR487333 | 1 | | 51% | | 363 | | Unknown protein | |  | |  | |  | |  | |
| GR487243 | 1 | | 52% | | 180 | | Unknown protein | |  | |  | |  | |  | |
| GR487383 | 1 | | 49% | | 195 | | Unknown protein | |  | |  | |  | |  | |
| GR487402 | 1 | | 31% | | 126 | | Unknown protein | |  | |  | |  | |  | |
| GR487439 | 1 | | 50% | | 336 | | Unknown protein | |  | |  | |  | |  | |
| GR487386 | 1 | | 53% | | 180 | | Unknown protein | |  | |  | |  | |  | |
| **Unknown conserved protein** | | | | | | | | | | | | | | | | |
| GR487373 | 1 | | 53% | | 135 | | Unknown conserved protein | | 4.0e-07 | | 51 | | agambiae.EST-CLIPPED.mar08.fa | | CD743825.1 | |
| GR487350 | 4 | | 54% | | 339 | | Unknown conserved protein | | 6.0e-12 | | 69 | | agambiae.EST-CLIPPED.mar08.fa | | BM590044.1 | |
| GR487330 | 1 | | 54% | | 135 | | Unknown conserved protein | | 2.0e-08 | | 49 | | anopheles_darlingi_EST.fasta | | DV729374 | |
| GR487415 | 2 | | 57% | | 141 | | Unknown conserved protein | | 8.0e-13 | | 72 | | agambiae.EST-CLIPPED.mar08.fa | | BX006650.1 | |
| GR487397 | 1 | | 58% | | 189 | | Unknown conserved protein | | 5.0e-06 | | 48 | | agambiae.EST-CLIPPED.mar08.fa | | BX766947.1 | |
| **Bacterial protein** | | | | | | | | | | | | | | | | |
| GR487187 | | 3 | | 55% | | 78 | | Bacterial protein | | 2.0e-08 | | 57 | | refseq_protein | | ZP_00630616 |
| GR487196 | | 10 | | 59% | | 87 | | Bacterial protein | | 2.0e-08 | | 57 | | refseq_protein | | ZP_00630616 |
