# Supplementary material for: Anopheles aquasalis Infected by Plasmodium vivax Displays Unique Gene Expression Profiles when Compared to Other Malaria Vectors and Plasmodia
Source: PLoS One. 2010 Mar 22;5(3):e9795. doi: 10.1371/journal.pone.0009795 (PMC2842430; doi:10.1371/journal.pone.0009795)
Supplement: Table S4 — List of sequences from the 24 hours infected minus non-infected insects library. Sequences with significant similarity on BLASTN or BLASTX were grouped based on the function of the homologous protein.Sequences with significant similarity on BLASTN or BLASTX were grouped based on the function of the homologous protein. (0.12 MB DOC) [file pone.0009795.s006.doc]

| **Accession number** | **Number of reads** | **G+C Content** | **CDS Length** | **Annotated Description** | **E-value** | **Score** | **Organism/Database** | **Gene**  **Accession**  **no.** |
| --- | --- | --- | --- | --- | --- | --- | --- | --- |
| **Signal transduction mechanism** | | | | | | | | |
| GR487536 | 1 | 56% | 261 | Rhodopsin receptor 1 | 5.0e-45 | 174 | Aedes_aegypti.AaegL1.50.pep.all.fa | AAEL006498-PA |
| **Biomolecules degradation** | | | | | | | | |
| GR487853 | 1 | 49% | 102 | Chymotrypsin 1 | 9.0e-14 | 60 | anopheles_aquasalis_ptna.fasta | AAD17491 |
| **Replication, translation and transcription** | | | | | | | | |
| GR487475 | 1 | 48% | 258 | Ribosomal protein S2 | 1.0e-124 | 450 | aaegypti.CONTIGS-Liverpool.AaegL1.fa | AAGE02025428.1 |
| GR487585 | 1 | 48% | 258 | Ribosomal protein S2 | 1.0e-124 | 450 | aaegypti.CONTIGS-Liverpool.AaegL1.fa | AAGE02025428.1 |
| GR487592 | 1 | 47% | 645 | 18S small subunit ribosomal RNA | 1.0e-141 | 491 | anopheles_darlingi_nucleotideo.fasta | AF417770 |
| GR487676 | 1 | 47% | 663 | 18S small subunit ribosomal RNA | 1.0e-148 | 515 | anopheles_darlingi_nucleotideo.fasta | AF417770 |
| GR487656 | 1 | 48% | 273 | 18S small subunit ribosomal RNA | 1.0e-119 | 419 | anopheles_darlingi_nucleotideo.fasta | AF417770 |
| GR487750 | 1 | 49% | 516 | 18S small subunit ribosomal RNA | 1.0e-112 | 394 | anopheles_darlingi_nucleotideo.fasta | AF417770 |
| **Defense and detoxification** | | | | | | | | |
| GR487564 | 1 | 61% | 150 | Bacteria responsive protein 2 / imaginal disc growth factor | 5.0e-22 | 98 | Anopheles_gambiae.AgamP3.50.pep.all.fa | AGAP008060-PA |
| **Energy metabolism** | | | | | | | | |
| GR487489 | 1 | 55% | 636 | Diacylglycerol acyltransferase | 1.0e-120 | 405 | Anopheles_gambiae.AgamP3.50.pep.all.fa | AGAP005949-PB |
| **Embryogenesis** | | | | | | | | |
| GR487801 | 2 | 55% | 381 | Vitellogenin | 1.0e-44 | 181 | uniref90.fasta | UniRef90_Q49MF2 |
| **Transport and secretion** | | | | | | | | |
| GR487840 | 1 | 53% | 228 | Thiamine transporter 1 / folate transporter | 2.0e-29 | 122 | cpipiens.PEPTIDES-CpipJ1.1.fa | CPIJ007516-PA |
| **Unknown protein** | | | | | | | | |
| GR487867 | 2 | 53% | 348 | Unknown protein |  |  |  |  |
| GR487535 | 1 | 41% | 768 | Unknown protein |  |  |  |  |
| GR487529 | 1 | 49% | 810 | Unknown protein of C2H2 and C2HC zinc fingers family |  |  |  |  |
| GR487904 | 1 | 41% | 159 | Unknown protein |  |  |  |  |
| GR487471 | 6 | 45% | 291 | Unknown protein |  |  |  |  |
| GR487568 | 1 | 44% | 273 | Unknown protein with coiled-coil domain |  |  |  |  |
| GR487720 | 1 | 44% | 273 | Unknown protein with coiled-coil domain |  |  |  |  |
| GR487706 | 1 | 44% | 270 | Unknown protein with coiled-coil domain |  |  |  |  |
| GR487511 | 1 | 46% | 294 | Unknown protein with coiled-coil domain |  |  |  |  |
| GR487588 | 1 | 47% | 294 | Unknown protein with coiled-coil domain |  |  |  |  |
| GR487770 | 1 | 44% | 273 | Unknown protein with coiled-coil domain |  |  |  |  |
| GR487755 | 1 | 46% | 294 | Unknown protein with coiled-coil domain |  |  |  |  |
| GR487722 | 1 | 46% | 294 | Unknown protein with coiled-coil domain |  |  |  |  |
| GR487687 | 1 | 46% | 288 | Unknown protein with coiled-coil domain |  |  |  |  |
| GR487476 | 1 | 45% | 291 | Unknown protein with coiled-coil domain |  |  |  |  |
| GR487640 | 1 | 46% | 291 | Unknown protein with coiled-coil domain |  |  |  |  |
| GR487595 | 1 | 44% | 273 | Unknown protein with coiled-coil domain |  |  |  |  |
| GR487714 | 1 | 46% | 270 | Unknown protein with coiled-coil domain |  |  |  |  |
| GR487566 | 1 | 46% | 291 | Unknown protein with coiled-coil domain |  |  |  |  |
| GR487602 | 1 | 44% | 273 | Unknown protein with coiled-coil domain |  |  |  |  |
| GR487649 | 1 | 45% | 273 | Unknown protein with coiled-coil domain |  |  |  |  |
| GR487668 | 1 | 42% | 252 | Unknown protein with coiled-coil domain |  |  |  |  |
| GR487683 | 1 | 46% | 288 | Unknown protein with coiled-coil domain |  |  |  |  |
| GR487516 | 1 | 45% | 294 | Unknown protein with coiled-coil domain |  |  |  |  |
| GR487699 | 1 | 46% | 291 | Unknown protein with coiled-coil domain |  |  |  |  |
| GR487820 | 1 | 54% | 147 | Unknown protein |  |  |  |  |
| GR487821 | 1 | 44% | 270 | Unknown protein with coiled-coil domain |  |  |  |  |
| GR487816 | 1 | 43% | 240 | Unknown protein |  |  |  |  |
| GR487825 | 1 | 46% | 294 | Unknown protein with coiled-coil domain |  |  |  |  |
| GR487828 | 1 | 44% | 225 | Unknown protein |  |  |  |  |
| GR487817 | 1 | 47% | 291 | Unknown protein with coiled-coil domain |  |  |  |  |
| GR487837 | 1 | 44% | 267 | Unknown protein with coiled-coil domain |  |  |  |  |
| GR487754 | 1 | 50% | 219 | Unknown protein |  |  |  |  |
| GR487791 | 1 | 51% | 306 | Unknown protein |  |  |  |  |
| GR487789 | 1 | 51% | 303 | Unknown protein |  |  |  |  |
| GR487696 | 145 | 52% | 498 | Unknown protein |  |  |  |  |
| GR487591 | 1 | 42% | 177 | Unknown protein |  |  |  |  |
| GR487805 | 1 | 42% | 285 | Unknown protein |  |  |  |  |
| **Unknown conserved protein** | | | | | | | | |
| GR487847 | 1 | 49% | 180 | Unknown conserved protein with coiled-coil domain | 2.0e-07 | 49 | Anopheles_gambiae.AgamP3.50.pep.all.fa | AGAP003939-PA |
| GR487846 | 1 | 41% | 141 | Unknown conserved protein | 2.0e-06 | 43 | cpipiens.EST-CLIPPED.mar08.fa | EV343992.1 |
| GR487908 | 1 | 46% | 247 | Unknown conserved protein | 2.0e-09 | 61 | agambiae.EST-CLIPPED.mar08.fa | BX035406.1 |
| GR487555 | 1 | 54% | 147 | Unknown conserved protein | 4.0e-06 | 29 | aaegypti.EST-CLIPPED.mar08.fa | DV322465.1 |
| GR487819 | 1 | 50% | 72 | Unknown conserved protein | 1.0e-10 | 51 | aaegypti.EST-CLIPPED.mar08.fa | DV321842.1 |
| GR487627 | 2 | 52% | 84 | Unknown conserved protein | 7.0e-06 | 46 | cpipiens.EST-CLIPPED.mar08.fa | EV304184.1 |
| GR487594 | 1 | 35% | 246 | Unknown conserved protein | 6.0e-06 | 34 | A.stephensi_EST.fasta | EX222236 |
| **Bacterial protein** | | | | | | | | |
| GR487921 | 1 | 57% | 228 | TonB-dependent vitamin B12 receptor | 0.0e+00 | 76 | PS00430 | TONB_DEPENDENT_REC_1 |
| GR487533 | 6 | 42% | 177 | Bacterial UDP-N-acetylglucosamine 1-carboxyvinyltransferase | 3.0e-14 | 77 | uniprot_sprot.fasta | Q8A681 |
| GR487618 | 1 | 39% | 174 | Bacterial protein | 2.0e-13 | 74 | uniprot_sprot.fasta | Q8A681 |
| GR487658 | 1 | 43% | 192 | Bacterial protein | 1.0e-07 | 60 | Rrna.fasta | AF478100 |
| GR487701 | 1 | 43% | 99 | Bacterial protein | 5.0e-11 | 70 | uniref90.fasta | UniRef90_B4WEV8 |
| GR487600 | 1 | 64% | 198 | Bacterial protein | 1.0e-47 | 192 | Rrna.fasta | DQ306696 |
| GR487733 | 1 | 57% | 87 | Bacterial protein | 6.0e-41 | 137 | Rrna.fasta | DQ306696 |
| **Accession number** | **Number of reads** | **G+C Content** | **CDS Length** | **Annotated Description** | **E-value** | **Score** | **Organism/Database** | **Gene**  **Accession**  **no.** |
