# Supplementary material for: Anopheles aquasalis Infected by Plasmodium vivax Displays Unique Gene Expression Profiles when Compared to Other Malaria Vectors and Plasmodia
Source: PLoS One. 2010 Mar 22;5(3):e9795. doi: 10.1371/journal.pone.0009795 (PMC2842430; doi:10.1371/journal.pone.0009795)
Supplement: Table S5 — Primers used for quantitative PCR. (0.03 MB DOC) [file pone.0009795.s007.doc]

| **Primer name** | **Primer code** | **Sequence (5' - 3')** | **Amplicon length** |
| --- | --- | --- | --- |
| Actin | ACTFwd | GATCTGGCATCACACCTTCTACAAT | 104bp |
|  | ACTRev | TCTTCTCACGGTTGGCCTTCGGGTT |  |
| BRP | BRPFwd | CAACAAGGCAGGTTACGTGAA | 141bp |
|  | BRPRev | ACATCCGATTACAGCCGATACTT |  |
| Carboxypeptidase | CPFwd | GTAACCCCTGCTCGGACACTT | 82bp |
|  | CPRev | GTCTTCACGAACGCAGCCAACGATT |  |
| Cecropin | CECFwd | TGAACTTCACGAAACTCTTCATTGT | 127bp |
|  | CECRev | AACACATTCCGACCCAGCTTTTCAA |  |
| Fibrinogen | FIBFwd | TGGTTGGGTTGTCATTCAGCAT | 118bp |
|  | FIBRev | ACGATCAAGACCAAGCCAGAAT |  |
| Serpin | SRPNFwd | TCGTGTCACCTGCCTAAAGGATAAT | 115bp |
|  | SRPNRev | GTCCGCAAAATCCATCGTCGTATCA |  |
| Chymotrypsin | SERPROTFwd | CCCGATGAACTGATGAAAATTGATA | 95bp |
|  | SERPROTRev | GCACAGATTTCCTTGCTCTCGTCA |  |
